# Statistical and neural network modeling of β-glucanase production by *Streptomyces albogriseolus* (PQ002238), and immobilization on chitosan-coated magnetic microparticles

**DOI:** 10.1186/s40643-025-00862-z

**Published:** 2025-04-10

**Authors:** Nourhan H. Elshami, Ghadir S. El‑Housseiny, Mahmoud A. Yassien, Nadia A. Hassouna

**Affiliations:** Department of Microbiology and Immunology, Faculty of Pharmacy, Ain Shams University, Organization of African Unity St., POB: 11566, Abbassia, Cairo Egypt

**Keywords:** Optimization, Immobilization, β-Glucanase, Response surface methodology, Neural network, Characterization

## Abstract

β-Glucanases are a series of glycoside hydrolases (GHs) that are of special interest for various medical and biotechnological applications. Numerous β-glucanases were produced by different types of microorganisms. Particularly, bacterial β-glucanases have the privilege of being stable, easily produced, and suitable for large-scale production. This study aimed for finding potent β-glucanase producing bacterial strains and optimizing its production. Soil samples from Egyptian governorates were screened for such strains, and 96 isolates were collected. The β-glucanase activity was qualitatively assessed and quantitatively measured using 3,5-dinitrosalicylic acid (DNS) method. The highest β-glucanase producing strain (0.74 U/ml) was identified as *Streptomyces albogriseolus* S13-1. The optimum incubation period and temperature, determined one-variable at a time, were estimated as 4 d and 45 ͦ C, respectively. Similarly, yeast β-glucan and beef extract were selected as the best carbon and nitrogen sources, with enzymatic activities of 0.74 and 1.12 U/ml, respectively. Other fermentation conditions were optimized through response surface methodology (RSM); D-optimal design (DOD) with a total of 28 runs. The maximum experimental β-glucanase activity (1.3 U/ml) was obtained with pH 6.5, inoculum volume of 0.5% v/v, agitation speed of 100 rpm, carbon concentration of 1% w/v, and nitrogen concentration of 0.11% w/v. This was 1.76-fold higher compared to unoptimized conditions. Using the same experimental matrix, an artificial neural network (ANN) was built to predict β-glucanase production by the isolated strain. Predicted β-glucanase levels by RSM and ANN were 1.79 and 1.32 U/ml, respectively. Both models slightly over-estimated production levels, but ANN showed higher predictivity and better performance metrics. The enzyme was partially purified through acetone precipitation, characterized, and immobilized on chitosan-coated iron oxide microparticles. The optimal pH and temperature for enzyme activity were 5 and 50 °C, respectively. The immobilized enzyme showed superior characters such as higher stability at temperatures 50, 60, and 70 °C compared to the free enzyme, and satisfactory reusability, losing only 30% of activity after 6 cycles of reuse.

**Graphical Abstract:**

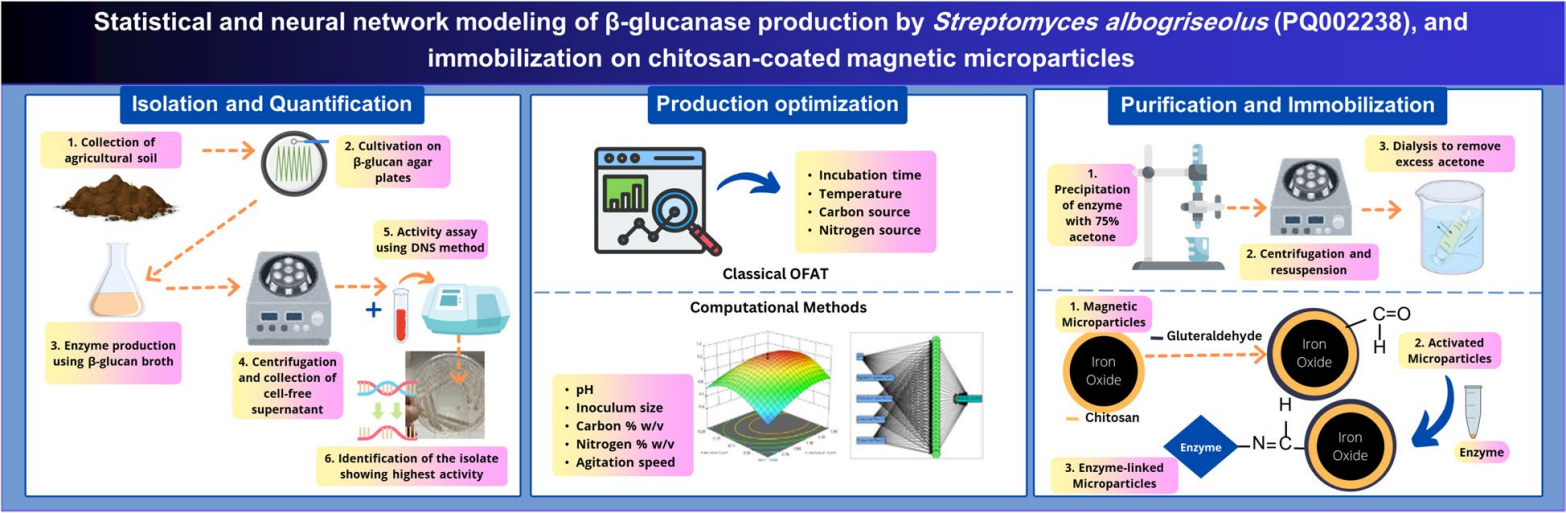

**Supplementary Information:**

The online version contains supplementary material available at 10.1186/s40643-025-00862-z.

## Introduction

Bioconversion is the process of transforming organic substrates into valuable products or energy sources through the action of biological processes or agents, such as microorganisms and enzymes. The immense presence of bioconversion processes in industrial biotechnology stems from the fact that they are eco-friendly, reducing environmental wastes and harnessing renewable resources to produce value-added products for applications in agriculture, food, pharmaceuticals, and biofuels (Siddiqui et al. 2023, 2025). Although many industrial processes have traditionally relied on the use of impure enzyme sources, usually animals or plants, the development of fermentation technologies has led to the commercial exploitation of microbial enzymes (Robinson 2015).

β-Glucans are naturally abundant polysaccharides that consist of glucose monomers connected via β-glycosidic bonds (Han et al. 2020). They have been identified in cereals, algae, seaweeds, and the cell walls of fungi and yeast, where they play vital functions (Nakashima et al. 2018). β-Glucanases, a series of glycoside hydrolases (GHs) that break down β-glucans into oligosaccharides and glucose, are among the most versatile enzymes that play a pivotal role in various industries. For example, their ability for effective deconstruction and elimination of antinutritional factors in plant-derived diets made them of great interest for the development of value-added food and feed products (Bampidis et al. 2024). Additionally, β-glucanases are used as a component in enzyme cocktails used for the saccharification of lignocellulosic biomass for biofuel production (Liu et al. 2024), inhibit the growth of fungi in fermentation technology, and are used in agriculture for tackling phytopathogenic fungi (Jiang et al. 2023). Medicinally, the biological activities of glucan-derived oligosaccharides with unique immunomodulatory, antitumor, and antibacterial activities have also drawn attention to the importance of finding novel β-glucanases capable of hydrolyzing β-glucans effectively (Chaudhari et al. 2021). Moreover, due to their activity against fungal biofilms, β-glucanases have been used in the treatment of dental fungal infections (Dukanovic Rikvold et al. 2024, Salah and Alwabsi 2024), and they have shown potential in modifying glucans for pharmaceutical purposes (Wang et al. 2024).

Microbial β-glucanases serve as attractive candidates for biotechnological applications, owing to their abundance, ease of isolation and manipulation, and stability (Jin et al. 2023)*.* Numerous β-glucanases have been isolated from a range of microorganisms including *Bacillus* sp., *Actinomycetes*, *Bacteroides*, *Clostridium* sp., *Streptococcus* sp., and *Pseudomonas* sp*.* (Jin et al. 2023)*. Streptomyces sp.*, which belong to the *Actinomycetes* order, are widely recognized for their notable capacity to produce a diverse array of industrial enzymes, including β-glucanases. Their remarkable metabolic versatility and ability to degrade complex polysaccharides makes them valuable for applications in bioconversion and biotechnology. (Barbuto Ferraiuolo et al. 2021; Lertcanawanichakul and Sahabuddeen 2023). Among these species, *Streptomyces albogriseolus* has been once reported for its production of stable β-1,3- and 1,4-glucanases (Van Zyl 1985); however, its enzymatic potential and properties remain largely unexplored. Given the growing demand for sustainable enzyme sources, further research into this strain could unlock novel insights in industrial enzyme production.

From a biotechnological point of view, production and stability optimization are vital for exploiting microbial enzymes in biotechnological processes. In enzyme production from bacteria, no single optimization approach is universally sufficient to achieve maximum yields. Each method has its merits: One-factor-at-a-time (OFAT) is simple and effective for preliminary screening, while statistical modelling offers a robust framework for understanding variables’ interactions and is cost-efficient. Despite being hardly interpretable, artificial neural networks (ANN), on the other hand, excel at capturing complex non-linear relationships in data, which is not always possible with conventional statistical methods (Singh et al. 2017). By combining these approaches, researchers can harness the strengths of each—starting with OFAT for initial screening, followed by response surface methodology (RSM) for precise optimization, and culminating with ANN for advanced modelling—ultimately leading to superior enzyme production outcomes.

Stability improvement, an important aspect of enzymes’ technical utility, can be achieved through enzyme immobilization, which is a widely studied technology for the enhancement of enzyme properties (Gregorio-Jauregui et al. 2012; Guisan et al. 2020a). Conventional immobilization methods such as adsorption, physical entrapment, and cross-linking have been widely studied for enzyme stabilization. However, these methods have several limitations, including low immobilization efficiency, susceptibility to inactivation, enzyme leakage, and resistance to mass transfer (Guisan et al. 2020b). Magnetic chitosan carriers have been recently explored as immobilization systems for several enzymes, overcoming the limitations of conventional carriers (Miri et al. 2024). Chitosan is a cationic polysaccharide with wide range of biological applications, owing to the presence of reactive amino and hydroxyl groups. It possesses unique properties such as biocompatibility, biodegradability, hydrophilicity, and mechanical stability, in addition to being inexpensive, rendering it suitable for large-scale applications (Kumari et al. 2018). Iron oxide microparticles are particles that consist of iron and oxygen atoms, owning unique magnetic and physicochemical characters, which allows their use as versatile carriers that can be effortlessly recovered from reaction vessels using a simple magnet (Mostafa et al. 2024).

In the present work, *Streptomyces albogriseolus S13-1*, obtained by screening soil samples from agricultural localities in Egypt, was capable of producing a moderately thermostable β-glucanase enzyme within an efficient time course. A combination of optimization strategies, namely OFAT, statistical modelling through RSM, and machine learning through ANN were used for production modelling and enhancement. To the best of our knowledge, this is the first work on ANN modelling of microbial β-glucanase production. Additionally, the produced β-glucanase was partially purified, characterized, and immobilized on chitosan-coated iron oxide magnetic microparticles, using glutaraldehyde as a coupling agent, which significantly enhanced its thermostability.

## Materials and methods

### Isolation and screening of β-glucanase producing bacteria

Soil samples were collected from several agricultural regions in Egypt. Samples were collected from a depth of 10–20 cm and sealed in sterile containers at 4 °C until use. Under aseptic conditions, 1 gm of soil sample was suspended in 100 ml of distilled water, agitated for 1 h, then serial dilutions of 10^–2^, 10^–3^, and 10^–4^ were prepared. After that, 0.1 ml of each concentration was spread on agar plates containing 0.5% water insoluble yeast β-glucan (Angel Yeast Co., China) as the sole carbon source, 0.05% K_2_HPO_4_, 0.05% KH_2_PO4, 0.1% Yeast extract, 0.05% MgSO_4_·7H_2_O, 0.1% (NH4)_2_SO_4_, and 1.2% Agar (Suyotha et al. 2017). The plates were incubated at 45 °C for 5 d, after which they were visually inspected. Colonies that had formed hydrolysis zones of ≥ 1.5 cm were isolated and kept at 4 °C on the same culture media. For long-term preservation, the isolates were maintained in Trypticase soy broth (TSB) containing 50% glycerol at − 80 °C.

### Preparation of crude enzyme for quantitative assessment of β-glucanase production

The isolates selected during initial screening were subjected to quantitative assessment of β-glucanase production. The shake-flask method was used for crude enzyme preparation. Fresh spore suspensions from 5-days old cultures (optical density, OD = 0.1) were inoculated (1% v/v) in 50 ml of the liquid production medium containing 1% soluble yeast β-glucan (Nanjing Tessin Biotechnology Co., Ltd., China), 0.05% KH_2_PO_4_, 0.05% K_2_HPO_4_, 0.1% Yeast extract, 0.05% MgSO_4_·7H_2_O, 0.001% FeSO_4_·7H_2_O, and 0.05% KCl (Suyotha et al. 2017), then were incubated for 120 h at 45 °C and 170 rpm. Samples were collected every 24 h, centrifuged (Sigma 3-18KS refrigerated benchtop centrifuge, Sigma Laborzentrifugen, Germany) at 10,000×*g* for 10 min at 4 °C, and the cell-free supernatants were used for enzyme activity assays. The pellets obtained from centrifugation were dried and weighed to monitor cell growth. The isolate with highest enzyme activity was selected for further study.

### β-Glucanase activity assay

β-Glucanase activity was determined using the 3,5-dinitrosalicylic acid (DNS) method described by Miller (1959) with some modifications. The reaction mixture contained 0.5% yeast β-glucan dissolved in phosphate-citrate buffer (pH 6.5), and a suitably diluted enzyme preparation. The mixture was incubated in a shaking water bath at 40 °C and 100 rpm for 60 min. The reaction was stopped by adding DNS reagent (1% DNS, 1.2% NaOH, 0.05% Na sulfite, 0.2% phenol, and 30% K Na tartarate), and heating in boiling water bath for 10 min followed by rapid cooling in an ice bath, then bringing back to room temperature. The absorbance was measured spectrophotometrically at 575 nm, using glucose as standard, and the enzyme activity was calculated according to Eq. 1 (Khalid et al. 2021). One unit of enzyme activity was defined as the enzyme amount that produces 1 μmol of reducing sugar per minute under the mentioned reaction conditions.

1 $$Units\;of\;enzyme\; activity\;\left( U \right) = X \cdot n/t/v$$

where X is the amount of reducing sugar produced (μ mol), n is the enzyme solution’s dilution factor, t is the reaction time (minutes), and v is the enzyme solution’s volume (ml).

### Morphological characterization and molecular identification of the selected β-glucanase-producing isolate

The cultural characteristics of the unknown isolate were visually inspected, and the mycelial connections were observed after Gram staining using an optical microscope with a magnification power of 40X. The phylogeny of isolate S13-1 was then established by sequence determination of the 16S ribosomal RNA gene. 16S ribosomal RNA amplification and sequencing were performed by Macrogen (South Korea). For PCR (polymerase chain reaction), the primer pair 27F (5ʹ-AGA GTT TGA TCM TGG CTC AG-3ʹ) and 1492R (5ʹ-TAC GGY TAC CTT GTT ACG ACT T-3ʹ) were used, while 785F (5ʹ-GGA TTA GAT ACC CTG GTA-3ʹ) and 907R (5ʹ-CCG TCA ATT CMT TTR AGT TT-3ʹ) were used for sequencing. The obtained sequence was then aligned and checked for homology using BLAST (Basic Local Alignment Search Tool: https://blast.ncbi.nlm.nih.gov/Blast.cgi) provided by NCBI GenBank. High similarity sequences were selected from GenBank and used for phylogenetic tree construction by Neighbor-joining statistical methodology (with 1000 bootstrap replications) using MEGA software v.11.0 (Kumar Lab, Temple University, Philadelphia, USA). The 16S rRNA sequence of the isolated strain was then deposited in NCBI GenBank, and a copy of the strain was preserved in the Culture Collection of Ain Shams University (CCASU), registered under the World Data Center for Microorganisms.

### Studying the effect of various fermentation conditions on β-glucanase production

For optimization of fermentation conditions, the spore count was adjusted before inoculation to ensure inoculum uniformity across all experiments. Using the method described by Samac et al. (2003), a standard curve that correlates the optical density (OD) at 600 nm to spore counts in colony forming units (CFU) was established by surface plating of serial dilutions from 5 d old spore suspension—prepared by adding distilled water and gently scratching the surface of a 5-d-old slant to dislodge the spores—(see supplementary file Figure S1). After adjustment of optical density to obtain a count of 1 × 10^8^ CFU/ml, an aliquot of 1% v/v of the prepared spore suspension was inoculated in 50 ml of the production media, and the crude enzyme was obtained as described before.

Preliminary experiments involved studying the effect of incubation time, temperature, type of carbon source, and type of nitrogen source on the levels of β-glucanase production by one-factor-at-a-time approach (OFAT). For the determination of optimum incubation time, *Streptomyces sp.* S13-1 was inoculated in β-glucanase production medium from 1 to 7 d at 45 °C. The enzyme activity was assayed at seven regular intervals of 24 h, and the bacterial growth was monitored by measuring dry cell weight at each interval. A temperature range (37 °C to 53 °C) that spans both below and above the expected optimum was tested to capture the full spectrum of enzyme production. Finally, to study the effect of changing the carbon source, production using barley flour, oat flour, corn flour, wheat flour, glucose, sucrose, and glycerol—instead of yeast β-glucan—was performed. Similarly, production using different nitrogen sources (yeast extract, beef extract, tryptone, malt extract, urea, and ammonium sulfate) in the media containing the best carbon source was also performed. The optimum condition of the four studied factors were fixed during further experiments.

### Statistical optimization of β-glucanase production using response surface methodology (RSM)

D-optimal design (DOD) was employed using five factors, three levels each, to understand the interactions of different fermentation variables, and to reach maximum β-glucanase production. The D optimal design is a computer aided design which sequentially adds and deletes points from a design to select an optimal combination of treatments from a set of possible runs for the experiment. The main advantage in D optimal design is that it is very flexible and can be used to fit any type of model and affords fewer number of experiments than classic Central Composite Designs and Box Behnken designs. The D-optimal design can be considered as the best design for generation of response surfaces (Ranade and Thiagarajan 2017). The factors and levels chosen are shown in Table 1. A total of 28 experiments were performed, including 3 replicates and 3 center points, which served to give a more accurate estimate of the overall process error and establish uniform precision. The response value—signaled as units of enzyme activity—was recorded as the mean of three readings for each experiment to minimize sample to sample variation. The data were fitted in second-order polynomial model that relates β-glucanase activity to independent fermentation variables, and the obtained model was then used to predict the optimal cultural conditions that yield maximum β-glucanase activity. For the statistical validation of the obtained model, analysis of variance (ANOVA) was used. The significance of the model was assessed using F-test, with a significance level of p = 0.05. The design of experiments and analysis of results were performed using Design Expert v. 13.0 (Stat-Ease Inc., Statistics Made Easy, Minneapolis, MN, USA).

**Table 1:** Variables and levels of D-optimal design (DOD)

Independent variable	Level		
	− 1	0	+ 1
pH (A)	6	7	8
Agitation speed (B, rpm)	100	170	240
Inoculum volume (C, % v/v)	0.5	1	1.5
Carbon concentration (D, % w/v)	0.5	1	1.5
Nitrogen concentration (E, % w/v)	0.01	0.11	0.2

### Artificial neural network analysis (ANN)

The ANN analysis was performed using the same experimental matrix and data that were used in RSM. A multi-layer feedforward architecture with one hidden layer was used. The input layer consisted of five nodes, representing the five independent variables under study, while the output layer consisted of β-glucanase activity as the response variable. The training was performed by supervised learning. A hyperbolic tangent activation function (Tan H) was used in the hidden layer, and hyperparameters—amely the number of hidden units and learning rate—were tuned by trial-and-error to obtain optimum results. For model validation, the k-fold cross-validation algorithm was used, where the dataset was divided into groups, part of which (k = 4) were used for training the model while the rest were used for validation, and all groups alternated between training and validation phases. At the end of the process a mean value was calculated from regrouped data to reflect an overall evaluation of the validation process (Xiong et al. 2020). To assess the fitness of the model, the coefficient of determination (R^2^), root mean square error (RMSE), mean absolute deviation (MAD), as well as the sum of squared errors (SSE) were evaluated, according to Eq. 2–5 (Ekpenyong et al. 2021). The desirability profiles were then used to predict β-glucanase production at the optimum conditions suggested previously by RSM. The ANN was developed and analyzed using JMP Pro v.17.0 (SAS Institute Inc., Cary, NC, 1989–2019).

2 $$R^{2} = 1 - \frac{{\mathop \sum \nolimits_{i = 1}^{n} \left( {y - \grave{y} } \right)^{2} }}{{\mathop \sum \nolimits_{i = 1}^{n} \left( {y - \overline{y}} \right)^{2} }}$$

3 $$RMSE = \sqrt {\frac{1}{n}\mathop \sum \limits_{i = 1}^{n} \left( {y - \grave{y}} \right)^{2} }$$

4 $$MAD = \frac{1}{n}\mathop \sum \limits_{i = 1}^{n} \left| {y - \grave{y} } \right|$$

5 $$SSE = \mathop \sum \limits_{i = 1}^{n} \left( {y - \grave{y} } \right)^{2}$$

where the number of samples is designated by n, the observed response value is designated by y, the predicted value is designated by ỳ, and the mean of the observed value (y) is designated by ӯ.

### Assessment of the prediction capacity of ANN in comparison to RSM model

The JMP Pro 17 comparison dialogue was used to compare the prediction capability of the two constructed models. The predicted and experimental data of the 28 runs in the experimental matrix were entered, then R^2^, RMSE, and MAD for both models were computed by the software. The mean percentage error (MPE) and mean absolute percentage error (MAPE) for both models were also determined using Eq. 6 and 7 (Ekpenyong et al. 2021):

6 $$MPE = \frac{1}{n}\mathop \sum \limits_{i = 1}^{n} \frac{{y - \grave{y} }}{y} \cdot 100$$

7 $$MAPE = \frac{1}{n}\mathop \sum \limits_{i = 1}^{n} \frac{{\left| {\grave{y} - y} \right|}}{\left| y \right|} \cdot 100$$

### Partial purification and size determination of β-glucanase enzyme

The crude enzyme preparation obtained through optimized fermentation conditions was precipitated using 75% acetone—previously chilled at − 20 °C—with gentle stirring at 4 °C for 2 h. The precipitate was recovered by centrifugation at 12,000×*g* for 15 min at 4 °C, resuspended in minimal volume of potassium phosphate buffer (0.05 M, pH 7). The suspension was then dialyzed against the same buffer for 24 h, with soft continuous stirring, and three changes of the dialysis buffer. The activity of the partially purified enzyme was then assayed as previously described, and the total protein content was assessed using Bradford’s method (Bradford 1976). The partially purified enzyme was stored at − 20 °C for future use.

The molecular weight and purity of the prepared β-glucanase was determined using sodium dodecyl sulfate polyacrylamide gel electrophoresis (SDS-PAGE), as described by Laemmli (1970), with 5% stacking and 12% separating gels. The protein samples were mixed with sample loading buffer containing 50 mM dithiothreitol (DTT) and heated for 2 min at 95 °C before loading to the gel. Following separation, the gel was stained using a solution of Coomassie Brilliant Blue (CBB) G-250 in 50% methanol and 10% glacial acetic acid, then destained by soaking in 40% methanol and 10% glacial acetic acid until bands were visible. The molecular weight of the protein was estimated by comparing its migration distance to a standard protein marker (10–250) (Genetix Puregene, USA) using GelAnalyzer software v.23.1.1 (available at www.gelanalyzer.com by Istvan Lazar Jr., PhD and Istvan Lazar Sr., PhD, CSc).

For zymogram analysis, the protein samples were mixed with the loading buffer without DTT or any reducing agent, then loaded without heating to 5% stacking gel and 12% separating gel containing 0.1% (w/v) yeast β-glucan. After electrophoresis, the gel was soaked in 50 mM phosphate buffer pH 6.5 containing 2.5% Triton X-100 for 30 min to remove the SDS. The gel was then incubated at 40 °C for 1 h in the same buffer without Triton X-100 to allow renaturation and enzymatic reaction. Finally, the gel was stained using 0.2% Congo red for 1 h and destained in 2M NaCl overnight until a clear degradation zone was observed (Champasri et al. 2015).

### Immobilization of β-glucanase on chitosan-coated iron oxide microparticles

A co-precipitation method was employed for the synthesis of iron oxide microparticles, along with simultaneous coating with a layer of low molecular weight chitosan (Mostafa et al. 2024). First, 0.25 g of soluble chitosan (Sigma Aldrich Co. LLS., Germany) was dissolved in 100 ml of distilled water. This was followed by the addition of 1 g of ferric chloride (FeCl_3_.6H_2_O) and 0.45 g of ferrous sulfate (FeSO_4_·7H_2_O). The solution was stirred continuously while 10 ml of 0.25% NH_4_OH were added dropwise until a black precipitate of chitosan coated iron oxide microparticles was formed. The formed particles were collected using an external magnet, washed several times with distilled water, and kept at 4 °C for further processing. Activation with glutaraldehyde was performed by mixing the formed microparticles (4 mg/ml) with 1% glutaraldehyde in phosphate buffer (0.05 M, pH 7) and incubation at room temperature for 3 h. Two ratios (5:1 and 10:1) for microparticles to glutaraldehyde were experimented. Finally, a solution of the partially purified enzyme with known activity was added to the glutaraldehyde activated microparticles in different ratios of 1:1, 2:1, and 3:1 respectively, and incubated at 4 °C for 6 h with continuous stirring at 180 rpm. To remove the excess glutaraldehyde and free enzyme, the microparticles were washed several times using 0.05 M phosphate buffer (pH 7), then resuspended in the same buffer and stored at 4 °C for characterization. The efficiency of the immobilization process was determined according to Eq. 8 (Muley et al. 2020):

8 $${\text{Immobilization efficiency }}\left( \% \right) = \frac{activity\;of\;the\; immobilized \;enzyme}{{activity \;of \;free \;enzyme}} \cdot 100$$

### Characterization of the β-glucanase loaded magnetic microparticles

To detect the structural changes associated with immobilization, Fourier-transform infrared (FTIR) spectra of chitosan-coated iron oxide particles, glutaraldehyde-activated particles, and enzyme loaded particles were obtained over a spectral range of 500–4000 cm^−1^ at room temperature. For the visualization of surface morphological changes, a scanning electron microscope (SEM) analysis was performed in the British University in Egypt (BUE). Samples were coated with a 10 nm conductive layer of gold, then observed with an acceleration voltage of 15 kV, and a working distance of 15–16 mm. The average particle size and particle size distribution of the enzyme-loaded particles were also determined using a dynamic light scattering (DLS) particle size analyzer.

### Measurement of enzyme reusability

The immobilized enzyme was mixed with 0.5% yeast β-glucan solution, and the mixture was incubated at 50 °C for 6 cycles, 1 h each. Following each cycle, an external magnet was used to collect the immobilized enzyme, and the substrate was replaced with fresh 0.5% β-glucan solution. The amount of reducing sugar present in solution after each cycle was assayed, and residual β-glucanase activity (%) was calculated.

### Effect of pH and temperature on enzyme activity and stability

To determine the optimal temperature for enzyme activity, the partially purified β-glucanase was allowed to react with 0.5% yeast β-glucan solution at various temperatures (30 °C–70 °C) and pH 6.5 followed by activity determination. For optimal pH, the enzyme activity on 0.5% yeast β-glucan was determined in 0.05 M of various buffers, namely citrate buffer (pH 3–5), phosphate buffer (pH 6–8), and glycine–NaOH buffer (pH 9) at 40 °C.

To compare the thermal stability of the free and immobilized β-glucanase enzyme, both enzyme solutions were preincubated at various temperatures (40 °C–70 °C) without substrate for a time interval that ranged from 10 min to 3 h, followed by the determination of residual enzyme activity under standard assay conditions. Similarly, the chemical stability was compared by measuring the residual enzyme activity of both the free and immobilized enzyme after preincubation for one h at different pH levels (4–9), followed by the standard assay procedure.

### Statistical analysis

All experiments in this study were conducted in triplicates, and data were recorded as mean ± standard deviation (SD). The obtained results were analyzed using one-way ANOVA followed by Tukey’s multiple comparison test. Results were considered statistically significant at *p* < 0.05. The analyses and graph construction were performed via GraphPad Prism v. 8.0.1 (GraphPad Software Inc., San Diego, CA, USA). For in-silico optimization experiments, the statistical analyses and graphical plots were obtained from Design Expert v. 13.0 or JMP Pro v.17.0, as mentioned previously.

## Results

### Isolation and screening for β-glucanase producing bacteria

From a total of 30 collected samples, 96 β-glucanase producing bacterial isolates were obtained at 45 °C during primary screening. Only 11 isolates showed a clear halo of ≥ 1.5 cm and thus were quantitively assessed for enzyme production. The bacterium that showed a zone of 1.7 cm and exhibited the highest activity (0.74 U/ml) on yeast β-glucan was isolate S13-1, which was selected for further study.

### Morphological characterization and molecular identification of isolate S13-1

After incubation for 5 d at 45 °C on β-glucan agar plates, the selected bacterium showed tough, small, opaque filamentous growth, with a sporulating brown surface and a characteristic earthy odor. The growing spores were initially observed as white, then progressed to grey, then brown according to the incubation time. Upon microscopical examination, the Gram-positive sporulated hyphae of *Streptomyces sp.* could be seen (see supplementary file Figure S2).

**Fig. 1:**
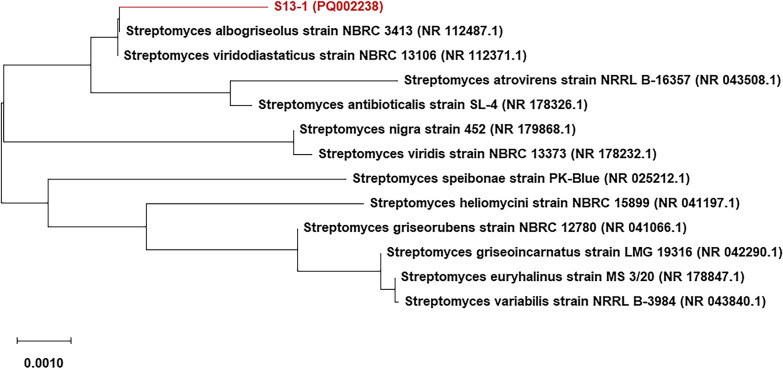
Phylogenetic tree showing the position of isolate S13-1 relative to its closest phylogenetic neighbors. The strain was identified as *S. albogriseolus* S13-1

The phylogenetic analysis revealed that the sequence of S13-1 showed 99.7% homology to the 16S ribosomal gene sequence of *Streptomyces albogriseolus* strain NBRC 3413 (NR_112487.1). The phylogenetic tree constructed using MEGA 11.0 software is shown in Fig. 1. Thus, the isolate was identified as *S. albogriseolus* S13-1, and its 16S rRNA sequence was submitted to NCBI GenBank under the accession number PQ002238. Additionally, the strain was deposited in the Culture Collection of Ain Shams University (CCASU) with the accession number CCASU-2024-70.

### Effect of incubation time, temperature, carbon source, and nitrogen source on β-glucanase production

The enzyme production in liquid yeast β-glucan medium was assessed daily for 7 d. The results showed that the optimum incubation period where highest enzyme activity and simultaneously highest bacterial growth could be seen was 4 d. The optimum incubation temperature was 45 °C, whereas a significant decline in enzyme activity was observed when temperature was reduced to 37 °C or raised to 53 °C. Given this data, the incubation time and incubation temperature were set to 4 d and 45 °C respectively during subsequent experiments (see supplementary file Figure S3 A&B).

Beside yeast β-glucan, the capability of S13-1 to utilize complex β-glucan containing carbon sources (1%), namely barley flour, oat flour, corn flour, and wheat flour was tested. The enzyme activity was highest with oat and corn flour, followed by barley flour, then wheat flour. Glucose, sucrose, and glycerol were not suitable for β-glucanase production from this strain. For further experiments, yeast β-glucan was chosen as the carbon source, since enzyme levels obtained with yeast β-glucan were significantly higher than other tested sources (see supplementary file Figure S3C).

Trials involving different nitrogen sources (0.1%) in the production medium were then performed, using yeast β-glucan as the sole carbon source. There was a non-significant difference in β-glucanase production from media containing tryptone, malt extract, urea, and ammonium sulfate in comparison with yeast extract, whereas the use of beef extract resulted in a significant increase in activity, thus it was used in the subsequent experiments (see supplementary file Figure S3D).

### Statistical optimization of β-glucanase production using response surface methodology (RSM)

The production of β-glucanase was optimized by implementing experiments following the D-optimal design. Table 2 displays the observed and forcasted values obtained from 28 runs. The variation in β-glucanase activities fluctuated between 0.47 and 1.27 U/ml. A second-order equation was obtained from the regression results of the performed experiments. As oberved in the Box-Cox plot (see supplementary file Figure S4A), a log transformation of the response variable was suggested by the software, which was applied to obtain better results. The final equation is presented as Eq. 9, where Y is the predicted β-glucanase activity for the coded parameters A, B, C, D, and E:

9 $$\begin{aligned} \ln \left( Y \right) & = - {5}.{51457} + {1}.{\text{7175 A}} - 0.000{1}0{\text{9446 B}} - {2}.0{\text{8958 C}} + {1}.{\text{64812 D}} + {5}.{\text{48676 E}} \\ & \quad + 0.628995{\text{ AE}} - 0.00161038{\text{ BD}} + 0.571711{\text{ CD}} - 2.01207{\text{ DE}} - 0.133568{\text{ A}}^{2} + 0.659735{\text{ C}}^{2} \, \\ & \quad - 0.714966{\text{ D}}^{2} - 34.9908{\text{ E}}^{2} \\ \end{aligned}$$

**Table 2:** D-optimal design showing the experimental runs, mean experimental values, and mean predicted values for β-glucanase production by *S. albogriseolus* S13-1

Run order	pH (A)	Agitation speed (B)	Inoculum volume (C)	β-glucan concentration (D)	Beef extract concentration (E)	β-glucanase activity (U/ml)	
						Actual values	Predicted values
1	7	240	0.5	1.5	0.2	0.83	0.78
2	8	240	1.5	1.5	0.2	0.71	0.72
3	8	240	1.5	1.5	0.01	0.67	0.70
4	6	170	1.5	1.5	0.2	0.93	0.91
5	8	100	1.5	1.5	0.01	1.06	1.00
6	7	170	1.0	1.0	0.11	1.27	1.21
7	6	240	1.0	1.5	0.01	0.75	0.76
8	8	100	1.5	0.5	0.2	0.62	0.62
9	6	240	1.5	0.5	0.01	0.47	0.48
10	8	100	0.5	1.5	0.2	0.93	0.94
11	7	100	1.5	1.5	0.2	1.14	1.21
12	8	100	0.5	0.5	0.01	0.67	0.66
13	7	135	1.25	0.75	0.11	0.91	0.99
14	7	170	1.0	1.0	0.11	1.21	1.21
15	8	100	0.5	1.5	0.01	0.90	0.91
16	6	170	0.5	0.5	0.01	0.81	0.83
17	6	240	0.5	1.0	0.2	0.91	0.96
18	6	240	0.5	0.5	0.11	1.21	1.17
19	6	100	1.0	0.5	0.2	0.70	0.70
20	7	240	0.5	1.5	0.2	0.78	0.78
21	6	100	1.0	0.5	0.2	0.70	0.70
22	8	240	0.5	1.0	0.01	0.73	0.73
23	7	170	1.0	1.0	0.11	1.20	1.21
24	6	100	0.5	1.5	0.01	1.19	1.22
25	6	100	1.5	1.0	0.01	1.09	1.02
26	7	100	0.5	0.5	0.2	1.16	1.17
27	8	240	1.0	0.5	0.2	0.60	0.58
28	6.00	240	0.5	0.5	0.11	1.16	1.17

The statistical significance of each term and the overall significance of the suggested model was assessed using Fisher’s F-test, as shown in Table 3. The regression analysis elucidated a model F-value of 54.28, which implies the model was significant overall. All parameters under study were found to have a significant influence on β-glucanase production. In addition, the interactions between factors AE, BD, CD, DE, and the terms A^2^, C^2^, D^2^, and E^2^ were also significant (p-value < 0.05). The lack-of-fit was statistically insignificant (p-value = 0.079), and the determination coefficient (R^2^) was 0.98, which confirms the goodness of fit of the model, as it suggests that 98% of the variation in β-glucanase activity was due to the studied variables, and only 2% of changeability could not be explained by the model. Similarly, the adjusted and the predicted determination coefficients (adjusted R^2^ and predicted R^2^) were in satisfactory agreement (0.96 and 0.92 respectively), and the adequate precision was 25.87 (> 4), which indicates a reasonable signal-to-noise ratio, thus the suggested model may be reliably used to navigate the experimental space.

**Table 3:** ANOVA for for response surface reduced quadratic model for β-glucanase production optimization using D-optimal design

Source of variation		Sum of Squares	Degrees of freedom	Mean Square	F-value	p-value	Significance
Model		1.84	13	0.1418	54.28	< 0.0001	Significant
Linear effect	A	0.1337	1	0.1337	51.15	< 0.0001	
	B	0.2622	1	0.2622	100.37	< 0.0001	
	C	0.1532	1	0.1532	58.62	< 0.0001	
	D	0.3347	1	0.3347	128.11	< 0.0001	
	E	0.0409	1	0.0409	15.65	0.0014	
Interaction effect	AE	0.0478	1	0.0478	18.28	0.0008	
	BD	0.0419	1	0.0419	16.02	0.0013	
	CD	0.2392	1	0.2392	91.53	< 0.0001	
	DE	0.1103	1	0.1103	42.22	< 0.0001	
Quadratic effect	A^2^	0.0474	1	0.0474	18.15	0.0008	
	C^2^	0.0760	1	0.0760	29.09	< 0.0001	
	D^2^	0.0998	1	0.0998	38.21	< 0.0001	
	E^2^	0.1939	1	0.1939	74.21	< 0.0001	
Residual effect	Lack of fit	0.0319	9	0.0035	3.77	0.0789	Not significant
	Pure error	0.0047	5	0.0009			
Corrected total		1.88	27				
R^2^	0.9805						
Adjusted R^2^	0.9625						
Predicted R^2^	0.9179						
Adequate Precision	25.8736						

Key model diagnostic plots are depicted in supplemetary file Figure S4. The normal probability plot of residuals illustrates that the residuals are normally distributed, which suggests that the normality assumption is reasonably justified. In the plot of predicted versus actual it can be seen that the points align in close proximity to the 45-degree diagonal line, implying that the responses predicted by the model were consistent with the values obtained experimentally. Taken together, these two plots imply that the current model is satisfactory. The residuals versus run order plot displays no apparent trends, indicating that there was no time-related bias affecting the experiment.

**Fig. 2:**
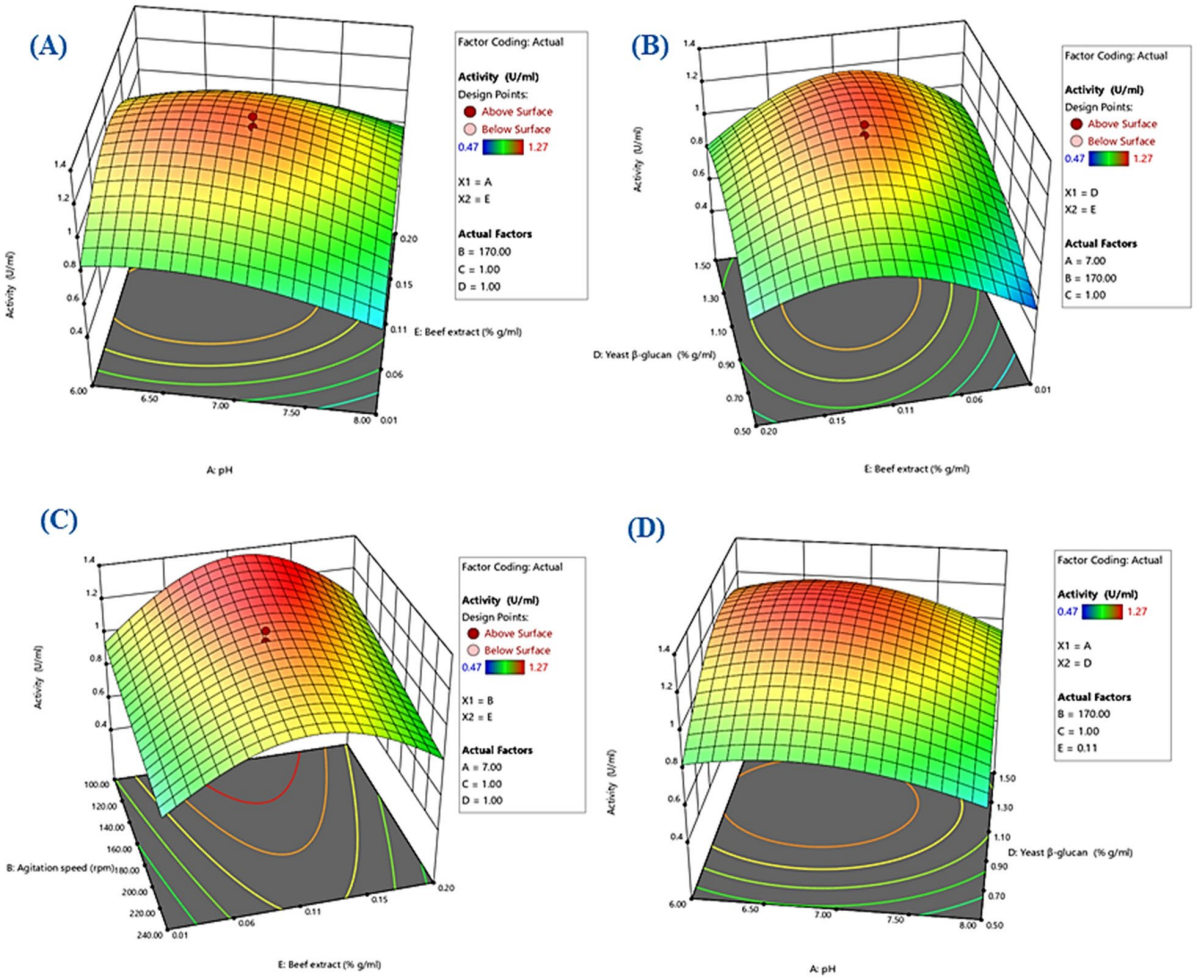
3D surface plots showing interactions between: **A** pH and beef extract concentration. **B** Yeast β-glucan and beef extract concentrations. **C** Agitation speed and beef extract concentration. **D** pH and yeast β-glucan concentration

To illustrate the interactions between independent variables, 3D surface plots (Fig. 2) and contour plots were constructed (see suplementary file Figure S5). Here, when the effect of two variables was plotted, the other variables were kept at central level. The elliptical nature of the 3D plots and the concentric contour lines of parameters AE, BD, CD, and DE conveyed the statistical significance of these interactions. It was observed that response variable (Y) peaks at moderate pH and nitrogen levels, whereas a gradual decrease is observed at lower and higher levels of these two parameters. Similarly, moderate carbon levels were found to be optimum for obtaining highest production in the presence of moderate nitrogen levels. In the presence of an average carbon level, smaller inoculum volume and lower agitation speed were favorable for the enzyme production, whilst larger inoculum volume and agitation speed resulted in lower production levels.

The RSM optimizer and the findings of 3D surface plots suggested production conditions of pH 6.5, 100 rpm, 1% w/v carbon, 0.11% w/v nitrogen, and 0.5% v/v inoculum volume for an optimal yield of β-glucanase enzyme. For model verification, *S. albogriseolus* S13-1 was cultured in the mentioned optimal conditions for 4 d at 45 °C, following by enzyme assay using DNS. The results showed that the measured value (1.3 U/ml) was nearly close to the model predicted response (1.79 U/ml).

### Artificial neural network analysis (ANN)

The most common multilayer feed-forward architecture with hyperbolic sigmoidal function was used to build a model that predicts the production of β-glucanase enzyme by *S. albogriseolus S13-1*, given the same input parameters used in RSM. The DOD matrix and the corresponding experimental responses were used to train the ANN. From all different topologies and combinations of ANN-specific hyperparameters, the ANN that showed maximum performance and best predictive capability had 20 neurons in the hidden layer (see supplementary file Figure S6). The neural network model was trained using a total of 200 epochs; this iterative process ensured that the model parameters were updated multiple times to improve the model’s generalizability. For boosting—an ensemble technique that combines multiple weak networks to create a stronger one—JMP software utilizes the Gradient Boosting to improve the model’s performance, and it was configured with 10 boosting iterations and a learning rate of 0.1 in the respective ANN. The random seed was set at a value of 1234 to ensure reproducibility.

The performance measures of the constructed network during both the training and validation phases are shown in Table 4. The model achieved an R^2^ value of 0.98 during the training phase, which suggests that the model adequately explains 98% of the data in the experimental space. The RMSE was 0.035, indicating that the models’ predictions deviate from the actual values by an average of 0.035 units, which implies model accuracy. To verify adequate performance, k-fold cross-validation with 4 folds was employed. The R^2^ and RMSE values averaged over all fold iterations were 0.95 and 0.05 respectively, which suggests that the model is performing well on unseen data indicating good generalization. The values of sum square error in both the training and validation sets were generally low (0.027 and 0.017 respectively), which conveys that the model is performing well in terms of minimizing the error between the predicted and actual response values. This is also shown in the predicted versus actual plot where all points lie close to the 45-degree line. The residual versus actual plot reveals that the residuals are randomly scattered around zero, suggesting that the model errors are random with no clear patterns or trends that suggest bias (see supplementary file Figure S7).

**Table 4:** Performance metrics of ANN and overall comparison of the predictive capability between DOD and ANN for β-glucanase production by *S. albogriseolus* S13-1 affected by the parameters under study

Measure	ANN		Model comparison		
	Training	Validation	Measure	ANN	RSM
R^2^	0.977	0.954	R^2^	0.972	0.944
RMSE	0.035	0.049	RMSE	0.04	0.056
MAD	0.025	0.032	MAD	0.027	0.033
SSE	0.027	0.017	MAPE	3.14	4.34
			MPE	0.5	1.98

*R*^*2*^ determination coefficient, *RMSE* root mean squared error, *MAD* mean absolute deviation, *SSE* sum squared error, *MAPE* mean absolute percentage error, *MPE* mean percentage error

The prediction profiler showed that the predicted value of β-glucanase activity at the optimal conditions of pH 6.5, 100 rpm, 1% w/v carbon, 0.11% w/v nitrogen, and 0.5% v/v inoculum volume was 1.32 U/ml which was highly comparable to the value obtained experimentally (see supplementary file Figure S8).

### The prediction capacity of ANN in comparison with RSM model

**Fig. 3:**
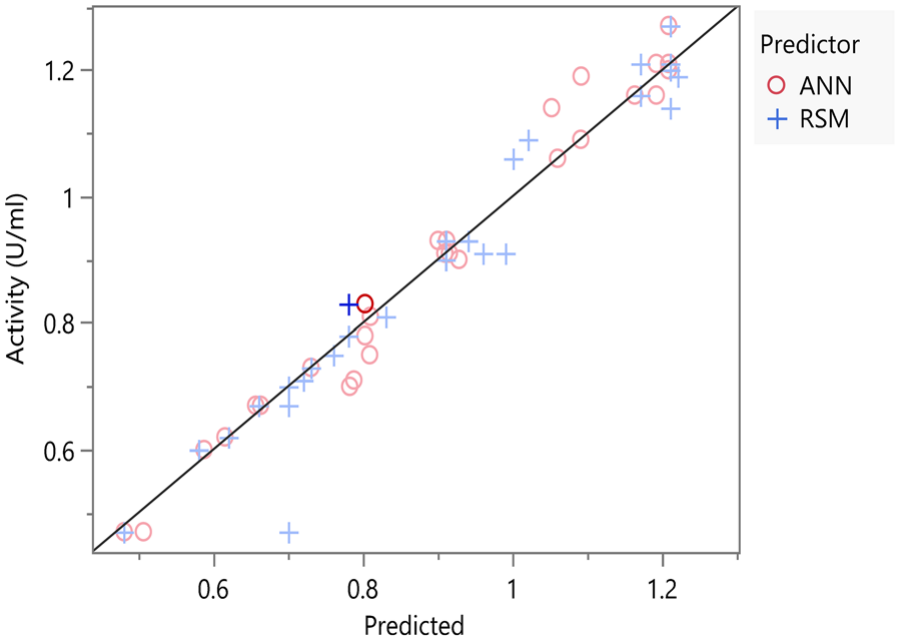
The Actual versus predicted plot of ANN and RSM models, constructed by JMP Pro 17.0 model comparison dialogue

The data shown in Table 4 compares the performance of both models based on the evaluation metrics obtained by JMP Pro 17 model comparison dialogue. The ANN model achieved a higher R^2^ value (0.97) compared to the RSM model (0.94), which indicates a better overall fit and explanatory power. Additionally, the ANN model demonstrated a slightly lower RMSE (0.04) compared to that of the RSM model (0.056). Other performance metrics such as MAD and MAPE also showed a slight outperformance of the ANN over the RSM model, with a lower percentage of error and better prediction power on average. The consistent patterns and low variance in the ANN’s predicted versus actual plot indicate good generalization ability, whilst the RSM model showed slightly higher variance suggesting that it may not capture some of the complexities in the data (Fig. 3).

Looking at the MPE of the two models, a slight overestimation bias could be seen. On average, the MPE of ANN model was quite small (0.5%), suggesting that the model has very little bias, whereas the MPE of the RSM model was 1.97% which is relatively low, but indicates that the RSM model has a higher tendency to overestimate the values of enzyme activity.

The outperformance of ANN over RSM was experimentally validated, where the highest value of β-glucanase production obtained at optimal conditions was 1.3 U/ml. This value is equivalently computed by the ANN (1.32 U/ml), whereas the theoretical value computed by the RSM model was higher than the actual (1.79 U/ml), implying the better predictive power of ANN over RSM.

### Partial purification and size determination of β-glucanase enzyme

The partial purification using 75% acetone resulted in 2.67-fold increase in specific activity. The specific activity before and after purification is shown in Table 5. Upon comparing SDS-PAGE and zymogram results, the molecular weight of the produced β-glucanase enzyme was approximated as 31 kDa (see supplementary file Figure S9). This size was computed by GelAnalyzer software, based on the migration distance of the protein band compared to the migration distances of standard protein marker.

**Table 5:** Comparison of the crude and partially purified enzyme preparations obtained from *S. albogriseolus* S13-1

Purification step	Volume (ml)	Total activity (U)	Purification Yield (%)	Total protein (mg)	Specific activity (U/mg)	Purification fold
Crude extract	1000	1330	100	49	27.14	1
Partially purified using 75% acetone	200	798	60	11	72.55	2.67

### Immobilization of β-glucanase on chitosan-coated iron oxide microparticles

To efficiently immobilize the β-glucanase enzyme on the prepared chitosan iron oxide particles, glutaraldehyde was used as the cross-linking agent. It was noticed that the amount of glutaraldehyde influenced the entrapment efficiency. A glutaraldehyde (1%) to microparticles ratio of 1:10 was most suitable for enzyme entrapment. On the contrary, increasing the amount of enzyme in the reaction mixture improved the immobilization efficiency. Upon activity determination, the highest immobilization yield (96.2%) was achieved with an enzyme to magnetic particles ratio of (3:1). Decreasing this ratio to (2:1), decreased the immobilization yield to 80%, while a ratio of (1:1) was not sufficient, and only resulted in a 10% entrapment. The magnetic property of the formed iron oxide particles was confirmed by observing the attraction of the particles to an external magnet (see supplementary file Figure S10). This magnetism was employed in the recovery of iron oxide particles from suspension.

### Characterization of the β-glucanase loaded magnetic microparticles

**Fig. 4:**
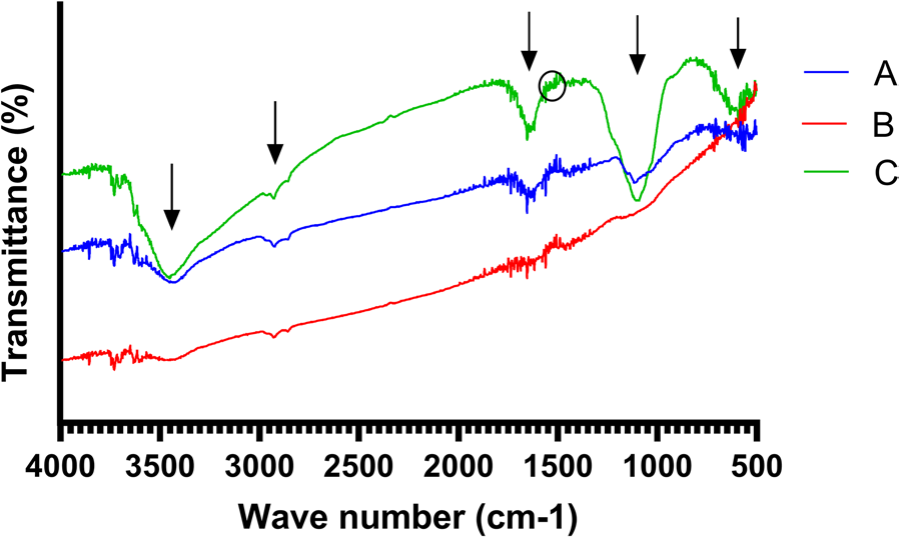
FTIR spectrum of **A** chitosan coated iron oxide microparticles, **B** glutaraldehyde-activated iron oxide microparticles, **C** enzyme-linked microparticles

The FTIR spectra were recorded to confirm the successful coating of iron oxide particles with chitosan, the activation with glutaraldehyde, and the subsequent enzyme loading. Results are illustrated in Fig. 4. The FTIR spectrum of chitosan functionalized particles showed a broad peak ~ 3494 cm^−1^, which corresponds to the O‒H stretching (Fig. 4A). Peaks observed at ~ 2923 cm^−1^, ~ 1617 cm^−1^, and ~ 1112 cm^−1^ can be attributed to the C–H stretching vibrations, vibrational modes of amide group (amide I band), and the C–O stretching vibrations, respectively. This confirms the successful attachment of chitosan on the magnetic particles. The peak at ~ 570 cm^−1^ is attributed to the Fe–O band of the magnetic support. The successful bonding of glutaraldehyde to chitosan at the cross-linking step is confirmed by reduction in the intensity of N–H vibrational bands of chitosan, along with the appearance of –C=N (imine) stretching band at ~ 1654 cm^−1^ (Fig. 4B). Additionally, a peak at ~ 1735 cm^−1^ revealed the presence of free –CHO of glutaraldehyde. Finally, the FTIR spectrum of the enzyme-linked particles revealed the presence of amide I and II bands at ~ 1651 cm^−1^ and ~ 1545 cm^−1^, respectively, along with a broad N–H band at ~ 3482 cm^−1^, which verifies the successful anchoring of the enzyme to the magnetic particles. The prominent C–O peak at ~ 1092 cm^−1^ indicates the presence of free oxygenated functional groups of the linked β-glucanase (Fig. 4C).

**Fig. 5:**
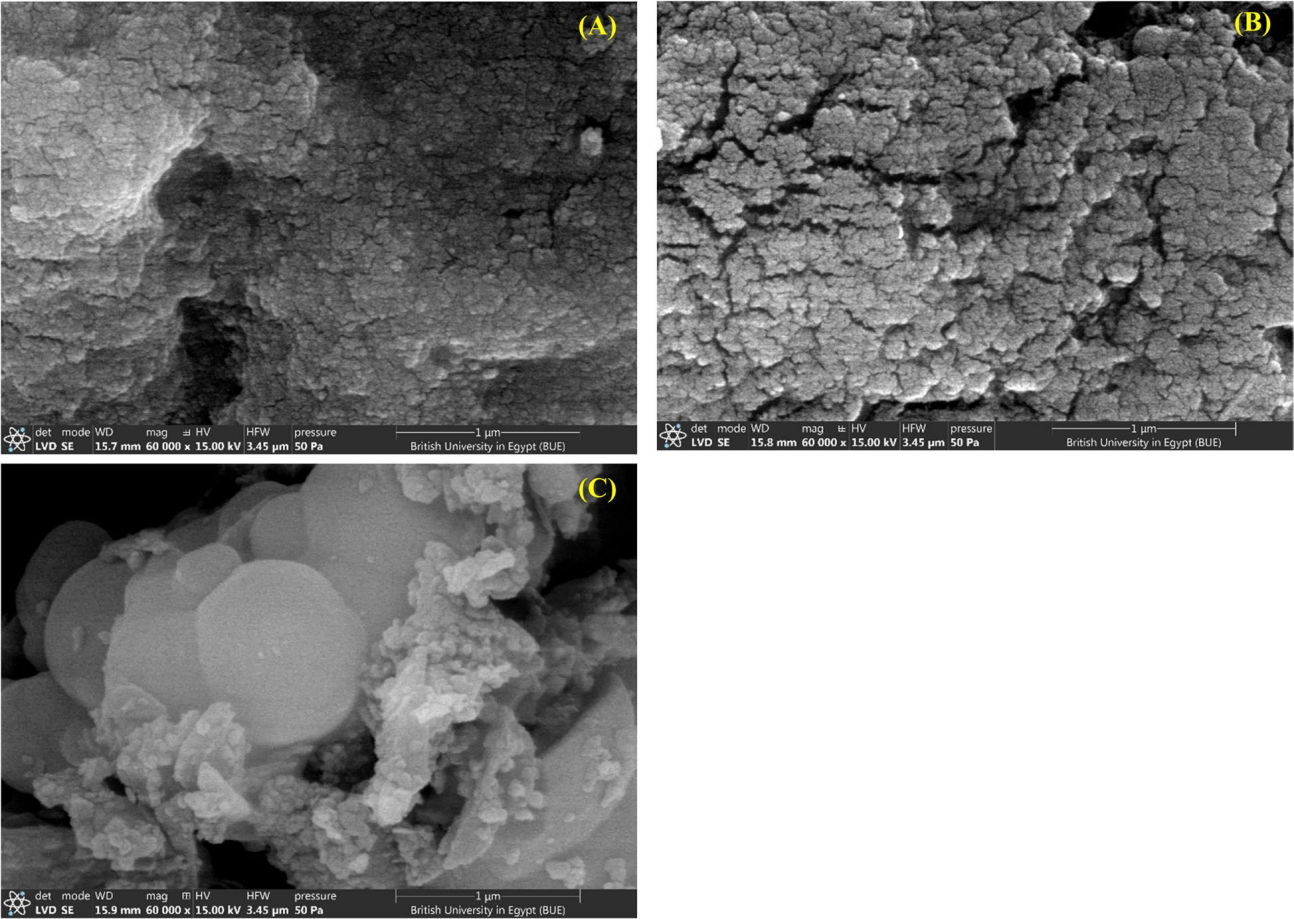
SEM images of **A** chitosan coated iron oxide microparticles, **B** glutaraldehyde-activated iron oxide microparticles, **C** enzyme-linked microparticles

Comparing the SEM images of chitosan-coated particles to the glutaraldehyde treated particles, it was observed that irregularities and surface roughness were introduced (Fig. 5A and B). Textural heterogeneity suggested the formation of cross-linked networks of glutaraldehyde with chitosan on the particles’ surface, which resulted in a more rugged appearance compared to the smooth surface of particles before glutaraldehyde addition. Upon enzyme immobilization, SEM analysis revealed new granular structures appearing as white patches on the particles surface, which can be attributed to the physical presence of the immobilized enzyme (Fig. 5C).

The DLS analysis of the enzyme loaded chitosan iron oxide particles showed an average hydrodynamic diameter of 1233 nm and a polydispersity index (PDI) of 0.45. The relatively high PDI indicates a moderately polydisperse system, which could be attributed to variations in the extent of surface modifications during the enzyme attachment process.

### Measurement of enzyme reusability

**Fig. 6:**
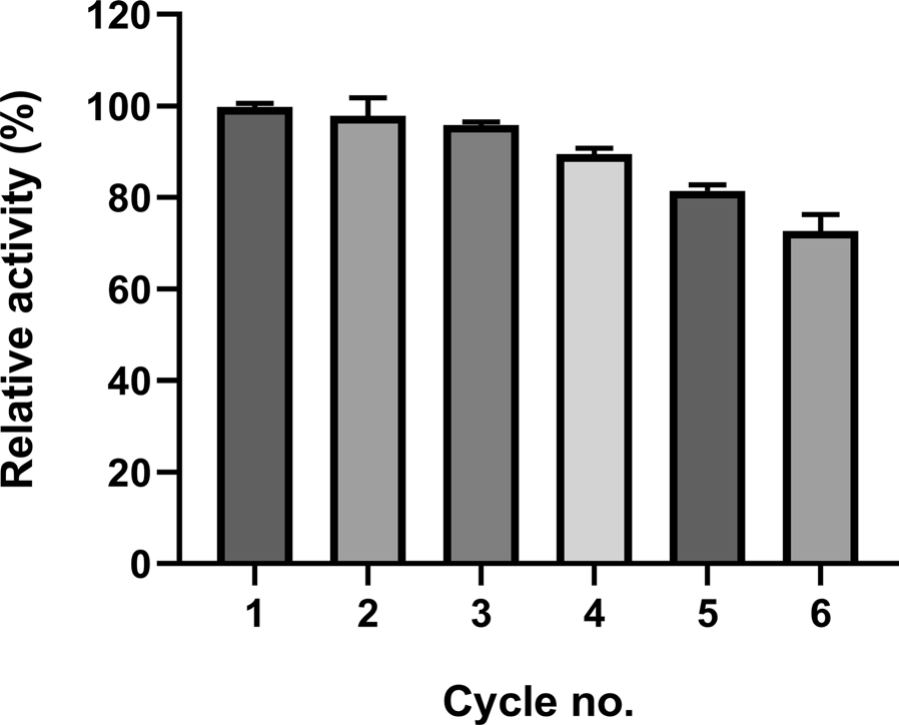
Reuse of β-glucanase enzyme from *S. albogriseolus* S13-1 over six cycles. Vertical bars are displayed as mean ± standard deviation

The recyclability of the immobilized enzyme was investigated over the course of 6 sequential cycles. As shown in Fig. 6, the residual activity slowly decreased to 72. 74% after the 6th cycle.

### Effect of pH and temperature on enzyme activity and stability

**Fig. 7:**
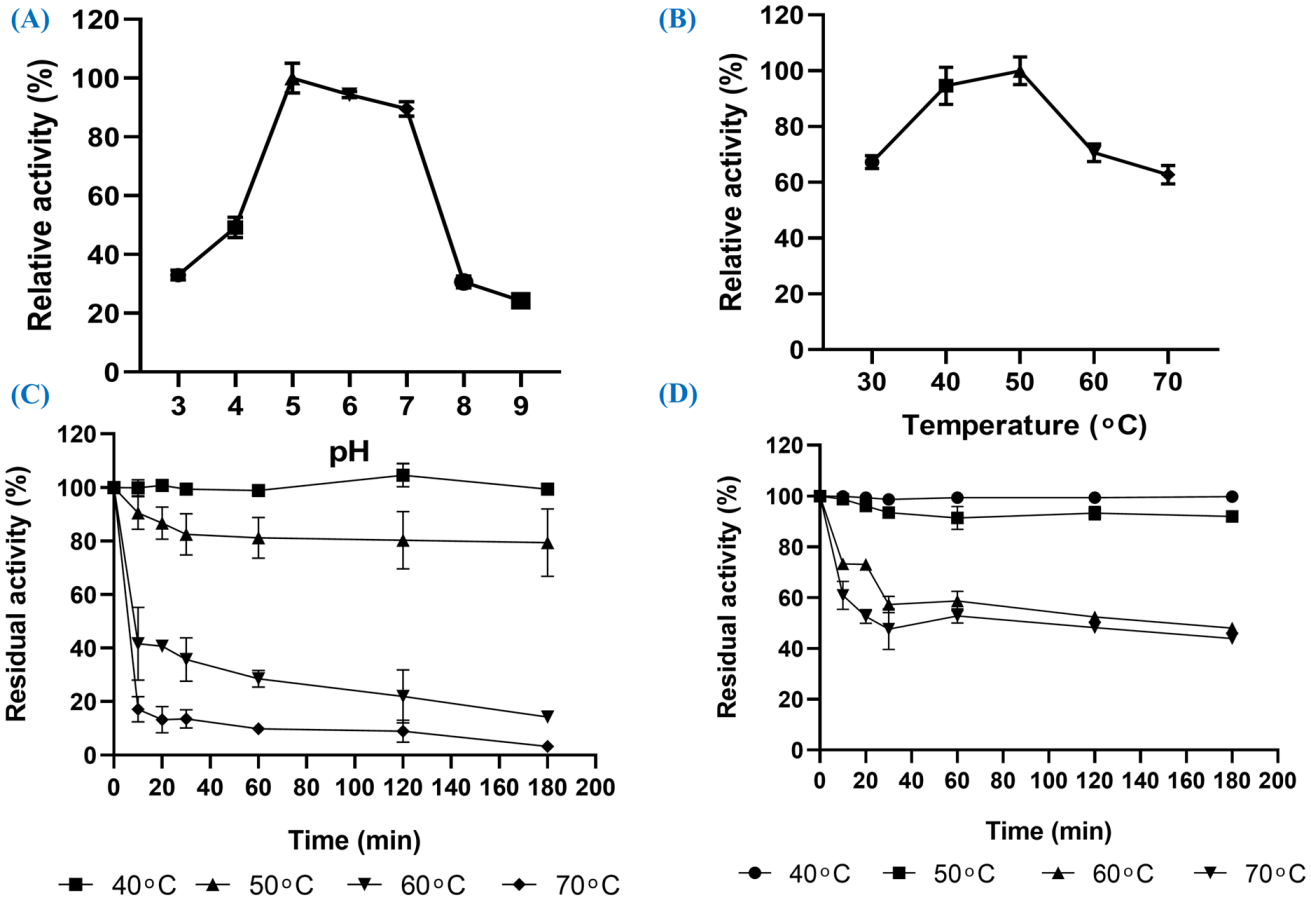
**A **Effect of pH on β-glucanase activity, **B** effect of temperature on β-glucanase activity, **C** thermal stability of free β-glucanase, **D** thermal stability of immobilized β-glucanase

The β-glucanase enzyme isolated from *S. albogriseolus* S13-1 exhibited activity over a broad pH range (3–9). The enzyme activity reached its maximum value at pH 5, followed by a non-significant decrease at pH 6. At pH 3 and pH 9, the enzyme retained almost 33% and 24% of its optimal activity, respectively (Fig. 7A). Additionally, the enzyme was stable over the same pH range, keeping more than 97% of its initial activity after incubation for 60 min, with no statistical difference in enzyme stability between the tested pH values.

The activity over a temperature range of 30–70 °C was examined. The temperature at which β-glucanase showed the highest activity was 50 °C, but this value was not statistically significant from the activity level at 40 °C (Fig. 7B). The enzyme retained about 63% of this optimal activity when the temperature was raised to 70 °C, whilst at 30 °C the activity was about 67% of the maximum value. Upon stability evaluation, the free enzyme was found to be stable at 40 °C, and retained 79% of its activity after 3 h of incubation at 50 °C. The activity dropped to about 14% after the 3 h course of incubation at 60 °C and to 3% after the same incubation period at 70 °C (Fig. 7C).

In contrast to the stability profile of the free enzyme at 40–70 °C, the stability of the immobilized enzyme was relatively higher, retaining 92% of its initial activity after 3 h of incubation at 50 °C, and as high as 48% and 44% of its initial activity at 60 and 70 °C (Fig. 7D). No significant difference was observed in the chemical stability (pH 3–9) of the immobilized enzyme compared to the free enzyme.

## Discussion

β-Glucanases are a group of glycoside hydrolases capable of degrading β-glucans to glucose and β-oligosaccharides. Among all β-glucanases, those that are active against fungal and yeast β-glucans are required for their various industrial and medicinal utilities yet are still understudied. The present study reports the exploitation of *Streptomyces albogriseolus* S13-1 isolated from agricultural soil to produce β-glucanase capable of degrading yeast β-glucan. In 1985, Van Zyl reported the ability of *Streptomyces albogriseolus* strain C2-221 to produce 1,3- and 1,4-β-glucanases. Several studies later reported the ability of the *Actinomycetes* to produce different types of β-glucanases (Fayad et al. 2001; Hong and Meng 2003; Grigorevski de Lima et al. 2005; Cecchini et al. 2018; Wonglom et al. 2019; Edison and Pradeep 2020).

The enhancement of fermentation conditions for optimum production levels is a pivotal concept in enzyme industry. In this study, a combination of classical single-variable-at-a-time experiments (OFAT), statistically designed optimization experiments, and machine learning was performed (Singh et al. 2017). The preliminary OFAT experiments revealed that β-glucanase production peaked after 4 d of *S. albogriseolus* S13-1 incubation in liquid media containing yeast β-glucan. Nevertheless, vegetative growth and enzyme production were observed starting from the first 24 h of incubation. The optimum incubation temperature was found to be 45 °C. Lower enzymatic activity below this temperature could be attributed to insufficient energy, while the activity decline at higher temperatures could be caused by enzyme denaturation (Khalid et al. 2021; Ibrahim et al. 2021). These findings classify the currently reported strain as fast-growing moderately thermophilic strain (Chen and Qin 2011).

The type of carbon and nitrogen sources present in the production media have a considerable impact on metabolites production, thus were also selected during the initial OFAT experiments. The use of pure yeast β-glucan as the sole carbon source in the culture medium resulted in the highest -glucanase production (0.74 U/ml) and was used during subsequent experiments. Cereal-based flour, namely oat, corn, barley, and wheat resulted in lower production levels. Such complex β-glucan sources contain mixed polymers and mixed linkage β-glucans (Jin et al. 2023), which may have diverted the metabolic focus from producing specific β-glucanase. However, if production cost is a limitation, using cereal-based β-glucan containing flour might be a suitable alternative to the purified yeast β-glucan. Glucose was included in this study to aid in establishing a baseline for enzyme expression, where it provided a non-inducing condition in comparison to the more complex β-glucan-containing substrates. Unlike glucose, glycerol is considered a non-repressive carbon source that supports microbial growth while minimizing catabolite repression and enhancing secondary metabolite yields, and thus was also included in this study for comparison (Barbuto Ferraiuolo et al. 2021). The significantly low β-glucanase production using these carbon sources suggests that the enzyme is highly inducible rather than constitutive, which is consistent with previous findings (Tang et al. 2004). This shows that the inclusion of specific inducers is crucial for β-glucanase production. Regarding the best nitrogen source, the use of beef extract resulted in a significant improvement in β-glucanase production (1.12 U/ml). Previous work has reported a similar result for β-1,4-glucanase production from *Streptomyces sp*. (Budihal et al. 2016).

Statistical methods for process optimization are especially efficient, and economically superior to OFAT when it comes to studying a multi-variable system as in microbial enzyme production (Singh et al. 2017). Particularly, the RSM is a robust mathematical approach that uses statistical experimental designs and a set of constrained regression equations to seek the best process conditions (Box and Wilson 1951). Here, it was employed to study the influence of initial pH, inoculum volume, agitation speed, carbon concentration, and nitrogen concentration on β-glucanase production from the isolated strain. It is often recommended that process optimization experiments be performed sequentially, starting with a screening design and followed by additional experiments to fit a response surface. In many situations, however, when the number of factors is large and time or budget is limited, it is recommended that “one step screening optimization experiments” be used: experiments used simultaneously for screening and optimization (Lawson 2003). The D-optimal design was used in this study to create the experimental space, as it is one of the most accurate methods for statistical design of experiments (El-Housseiny et al. 2021). Three levels were assigned to each of the five factors based on previous studies (Budihal et al. 2016; Suyotha et al. 2017; Ruiwen et al. 2023), and OFAT results, and a total of 28 runs were conducted to investigate the influence of these factors on β-glucanase production. Multiple regression analysis (ANOVA) was used to evaluate the significance of each factor and the sources of variation within the experiment (El-Housseiny et al. 2021; Shrestha et al. 2022). As shown in the results, the performance metrics of the developed second order model implied the adequacy and overall fit of the model. Additionally, all five variables showed a significant effect on β-glucanase production (p-value < 0.05), with beef extract concentration (factor E) having the least significant effect. One of the main privileges of statistical optimization methods is the ability to investigate the effect of variables interactions on a certain response, which is not possible when studying a single factor at a time (Singh et al. 2017). In this study, the interactions between factors AE, BD, CD, DE were significant for β-glucanase production and were observed by looking at the 3D surface plots. The oblong nature of these plots depicts how different levels of a certain variable affect the response in the presence of another variable, and therefore reflects variables interactions and optimum levels to obtain the highest response (Ghribi et al. 2012). Using these plots, along with desirability prediction tools, the optimum conditions for highest β-glucanase production were predicted to be pH 6.5, agitation speed of 100 rpm, yeast β-glucan concentration of 1% w/v, beef extract concentration of 0.11% w/v, and an inoculum volume of 0.5% v/v. Under these conditions, and at an incubation temperature of 45 °C and 4 d of incubation, the experimental yield was 1.3 U/ml, which was 1.76-fold higher compared to the unoptimized conditions. Tang et al. (2004) reported 1.5-fold increase in β-glucanase activity from *Bacillus subtilis* ZJF-1A5 through medium optimization using RSM. Similarly, Raza et al. (2019) reported 6.08- and 1.68-fold increase in endoglucanase and xylanase production from *Bacillus sonorensis* BD92, while El-Sersy et al. (2010) reported 1.6-fold increase in β-1,4-glucanase activity from *Streptomyces ruber*.

Machine learning is particularly useful when dealing with biological datasets. This is because biological data are often affected by large number of factors and are usually too complex for human analysis (Greener et al. 2022). One of the emerging and most effective methods for biological tasks is deep learning with ANN. Such models basically consist of so-called artificial neurons that take in any number of input variables, apply a mathematical function, and return output values, based on which the model is trained, and its predictive ability is improved. This behavior simulates the connectivity and learning behavior of neurons in human brain, which is why they are named this way (Batista et al. 2021; Greener et al. 2022). As far as our knowledge extends, this is the first report on the prediction of microbial β-glucanase production using ANN. The optimal topology for ANN structure was denoted as 5-20-1, consisting of 5 input neurons, one hidden layer with 20 neurons, and a single output neuron. The hidden layer employed a hyperbolic tangent activation function in all neurons. The fact that ANNs are trained by fitting the data into non-linear mathematical functions, allows them to capture complexities in the data, which is not always possible with linear regression models (Saber et al. 2023). The performance and error metrics of the ANN and RSM models built in this study showed that both models had high precision and adequacy in predicting β-glucanase production from given inputs. While both models nearly suggested the same optimal conditions, the predicted value for enzyme production from ANN was much closer to the actual experimental result, indicating superior predictive accuracy. This suggests that ANN was better able to model subtle non-linearities in the system. The comparison between both approaches demonstrates the robustness of the optimization process and shows how ANN can enhance predictive precision beyond traditional statistical models. Several studies have previously compared the efficiency of neural network modelling to statistical modelling for the optimization of production conditions of certain microbial metabolites (Pathak et al. 2015; Wei et al. 2017; Ekpenyong et al. 2021; Ousaadi et al. 2021; Saber et al. 2023; El-Naggar et al. 2024), which all reported that ANNs showed better performance than RSM models regarding generalization and predictive modelling ability. Although ANNs paradigms can handle non-linearity better than other models, ANNs have some limitations. One limitation is computational complexity due to many process iterations, which increases the computational time required for training and validation. Moreover, ANNs can be seen as a black box, where the influence of each factor in the model cannot be separately defined, unlike RSM models that are more interpretable when it comes to isolating and understanding certain factors in the process (Greener et al. 2022; Saber et al. 2023). Overall, combining the two methodologies for process optimization provides better understanding and simultaneously better predictions compared to using solely ANN or RSM.

Partially purified β-glucanase was obtained through precipitation with cold acetone at a concentration of 75%. Gadallah et al. (2023) previously reported that acetone was superior to ammonium sulfate for β-glucanase precipitation and obtained a purification fold of 2.39 at the precipitation step. In this study, a purification fold of 2.67 was obtained. Upon SDS-PAGE analysis, the partially purified enzyme revealed few protein bands, thus a zymogram was used to determine the band that possesses β-glucanase activity. Matching β-glucanase bands on SDS-PAGE and Zymogram gels revealed a size of 31 kDa, comparable to what was found by Kurakake et al. (2013). Further purification of the enzyme was not necessitated in this study but is sometimes crucial if highly pure enzyme is required.

Immobilization technology is a dynamic research area in industries that exploit enzymes, owing to the wide range of advantages the immobilized enzymes provide, such as operational stability and ease of recovery for reuse (Robinson 2015). There are several methods for enzyme immobilization (Robinson 2015; Guisan et al. 2020a). Among these methods, cross-linking via covalent bonding provides the strongest linkage between the enzyme and the immobilization support (Robinson 2015; Cho et al. 2018). In this study, partially purified β-glucanase has been successfully immobilized on chitosan coated iron oxide microparticles, with glutaraldehyde being used as a cross-linking agent. The successful immobilization of different enzymes using the same magnetic support was previously reported (Agrawal et al. 2020; Miri et al. 2024). Chitosan has exceptional properties and versatility in supporting and encapsulating biological agents. Its biocompatibility, biodegradability, and hydrophilicity make it an ideal candidate for biomedical, agricultural, environmental, and commercial applications. For example, it has been used for the formulation of edible films and coatings for the preservation of various foods products such as fruits, vegetables, dairy, and animal products, and it has shown utility for the encapsulation of several bioactive molecules such as folic acid, curcumin, and omega-3-rich oils (Díaz-Montes and Castro-Muñoz 2021; Castro-Muñoz et al. 2023). Additionally, when activated with glutaraldehyde, chitosan has shown potential for efficient enzyme immobilization (Gür et al. 2018; El-Shora et al. 2021). The incorporation of iron oxide magnetic cores, has further expanded its utility by enhancing mechanical strength, thermal stability, and magnetic properties for targeted applications, include enzyme immobilization. Both chitosan and iron oxide are safe, biocompatible, and biodegradable materials that do not raise concern if leaching occurs during industrial processes (Gregorio-Jauregui et al. 2012; Yeon et al. 2019). When glutaraldehyde is present, the overall performance of the immobilized system is enhanced since it strengthens the interaction between the polymer phase (chitosan) and the immobilized enzyme. Glutaraldehyde contains aldehyde groups that can form covalent bonds with the amino groups of both the chitosan and the enzyme, which results in the formation of stable Schiff base linkages (–C=N–) enhancing the structural integrity of the system (El-Shora et al. 2021; Eranda et al. 2024). The micro-ranged particles size, and magnetic property of iron oxide ensure ease of recovery and reuse which is economically favored (Miri et al. 2024). This immobilization technique was selected to improve the enzyme’s stability and reusability, due to enhanced mechanical strength and resistance to leaching, making it more suitable for industrial applications. As described in the results section, increasing the glutaraldehyde concentration resulted in lower entrapment of the enzyme. This might be because the presence of high glutaraldehyde concentration leads to extensive cross-linking with the active groups of the chitosan layer, which leaves no space for the enzyme to bind, thus immobilization efficiency is reduced. Additionally, the enzyme to support ratio was found to have a high influence on immobilization results, with higher efficiencies being associated with increased enzyme ratio. These findings agree with the results reported by El-Shora et al. (2021) and Miri et al. (2024). The successful enzyme binding was confirmed by measuring the enzymatic activity of the immobilized enzyme and visualizing the immobilization effect on the microparticles surface using FTIR and SEM imaging. Changes in the stretching and bending frequencies were observed following glutaraldehyde addition to the chitosan coated particles and following the addition of the enzyme. Additionally, surface changes were evident upon SEM imaging of the native microparticles, compared to glutaraldehyde-activated particles, and enzyme-immobilized particles. These results were consistent with Cho et al. (2018) and Miri et al. (2024), and implied success in cross-linking and enzyme immobilization steps. The magnetic property of the microparticles core was utilized for the effortless recovery of the immobilized enzyme from reaction mixtures using an external magnet, and covalent bonding ensured durability of the immobilized enzyme. This was expressed through efficient reusability, as the immobilized enzyme retained > 70% of its activity after 6 1-h interval cycles of subsequent reuse.

The influence of pH and temperature on the activity and stability of the partially purified β-glucanase enzyme was investigated. Findings revealed that the β-glucanase from *Streptomyces albogriseolus* S13-1 exhibited optimum activity at acidic pH (pH = 5), which is similar to most β-1,3-glucanases (Hong & Meng 2003; Shi et al. 2010; Woo et al. 2014; Kusaykin et al. 2017). Additionally, the enzyme showed high stability through a wide pH range. This property of the enzyme was also seen in the enzymes isolated by Qin et al. (2023), Jaafar et al. (2020), and Shrestha et al. (2022), and is beneficial for various industrial applications. The optimal temperature for activity was found to be 50 °C, comparable to the findings of Yi et al. (2018). However, the enzyme exhibited high relative activity (> 60%) at the temperature range 30 °C–70 °C, which allows its industrial exploitation at different operational temperatures without losing much of its β-glycosidic activity. Thermal stability experiments showed that the enzyme was stable at 50 °C but showed lower stability at 60 and 70 °C. Comparatively, the immobilized β-glucanase showed a significantly higher stability at 50, 60, and 70 °C. An increase in thermal stability was reported following immobilization using different techniques (Robinson 2015), and particularly improved stability was observed when covalent bonding was involved (Barbosa et al. 2013; Osuna et al. 2015; El-Shora et al. 2021; Miri et al. 2024). High thermal stability is a required characteristic for industrial enzymes, since high operational temperatures prevent microbial contamination and reduce viscosity inside reaction vessels, if such condition is required (Jaafar et al. 2020).

## Conclusion

This study successfully demonstrated β-glucanse production from *Streptomyces albogriseolus* (PQ002238), optimization of production conditions using classical, statistical, and machine learning approaches, and immobilization on chitosan-coated iron oxide microparticles for enhanced thermal stability and reusability. The findings highlight the potential for enhancing enzyme productivity and stability through advanced optimization and immobilization techniques. Future work should focus on scaling up the optimized fermentation conditions to pilot and industrial-scale bioreactors. This will enable a comprehensive evaluation of process efficiency, advancing the potential for large-scale applications. Investigating the long-term stability of the immobilized enzyme system for industrial use, particularly in bio-catalysis and bioconversion technologies is another important aspect. Additionally, exploring the use of agricultural and industrial waste materials as cost-effective substrates for β-glucanase production presents a sustainable and economically viable direction for further research, contributing to greener and more cost-efficient bioprocessing systems.

## Supplementary Information

Supplementary Material 1.

## Data Availability

All data generated or analyzed during this study are included in this published article and supplementary file.

## Abbreviations

ANN: Artificial neural network
ANOVA: Analysis of variance
BLAST: Basic Local Alignment Search Tool
CBB: Coomassie Brilliant Blue
CCASU: Culture Collection of Ain Shams University
CFU: Colony forming units
DLS: Dynamic light scattering
DNS: 3,5-Dinitrosalicylic acid
DOD: D-optimal design
DTT: Dithiothreitol
FTIR: Fourier-transform infrared
MAD: Mean absolute deviation
MAPE: Mean absolute percentage error
MPE: Mean percentage error
NCBI: National Center for Biotechnology Information
OD: Optical density
OFAT: One-factor-at-a-time
PCR: Polymerase chain reaction
PDI: Polydispersity index
RMSE: Root mean square error
RPM: Round per minute
RSM: Response surface methodology
SD: Standard deviation
SDS-PAGE: Sodium dodecyl sulfate polyacrylamide gel electrophoresis
SEM: Scanning electron microscope
SSE: Sum of squared errors
TSB: Trypticase soy broth
