# Supplementary material for: Statistical and neural network modeling of β-glucanase production by Streptomyces albogriseolus (PQ002238), and immobilization on chitosan-coated magnetic microparticles
Source: Bioresour Bioprocess. 2025 Apr 10;12(1):32. doi: 10.1186/s40643-025-00862-z (PMC11985864; doi:10.1186/s40643-025-00862-z)
Supplement: Supplementary file 1 — Supplementary Material 1. [file 40643_2025_862_MOESM1_ESM.docx]

**Supplementary material**

**Statistical and neural network modeling of β-glucanase production by *Streptomyces albogriseolus* (PQ002238), and immobilization on chitosan-coated magnetic microparticles**

**Nourhan H. Elshami^1^, Ghadir S. El‑Housseiny^1^, Mahmoud A. Yassien^1^ and Nadia A. Hassouna^1^**

^1^Department of Microbiology and Immunology, Faculty of Pharmacy, Ain shams University, Organization of African Unity St., POB: 11566, Abbassia, Cairo, Egypt

* Corresponding Author: Dr. Ghadir S. El-Housseiny, PhD

Address: Department of Microbiology and Immunology, Faculty of Pharmacy, Ain Shams University, Organization of African Unity St., POB: 11566, Abbassia, Cairo, Egypt.

E-mail: [ghadir.elhossaieny@pharma.asu.edu.eg](mailto:ghadir.elhossaieny@pharma.asu.edu.eg)

ORCID: <https://orchid.org/0000-0001-7454-4148>

Mobile: (002) 01001547800

**Figure S1.** Relationship between optical density at 600 nm and spore count (CFU/ml)


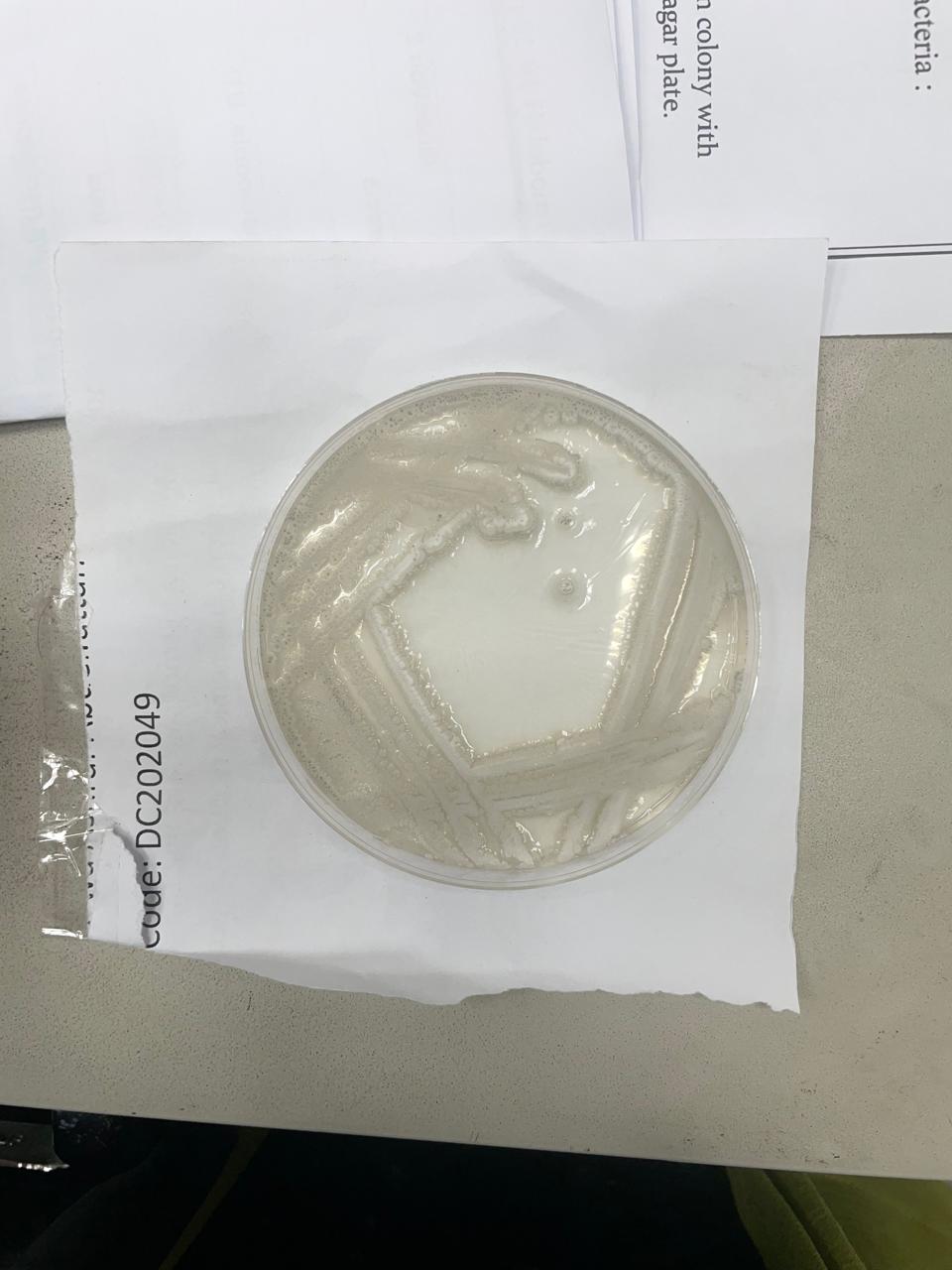


**A**


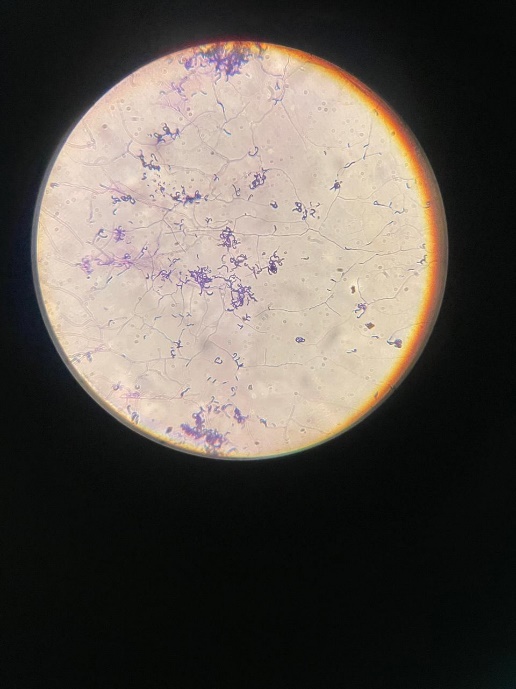


**B**

**Figure S2.** Colony morphology (A) and Gram-stained mycelium (B) of 5-days old culture of *Streptomyces sp.* S13-1 (x40)


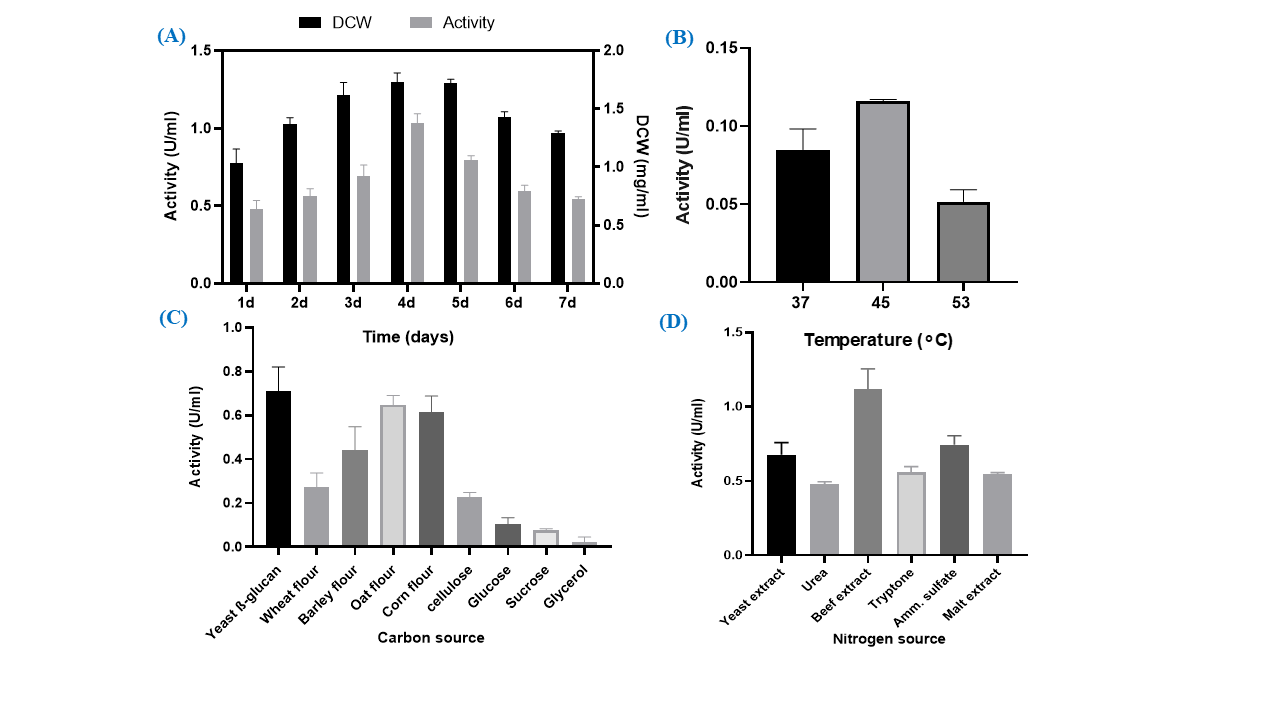


**Figure S3.** Effect of incubation time (days) **(A)**, temperature **(B)**, carbon source **(C)**, and nitrogen source **(D)** on β-glucanase production by *S. albogriseolus* S13-1.


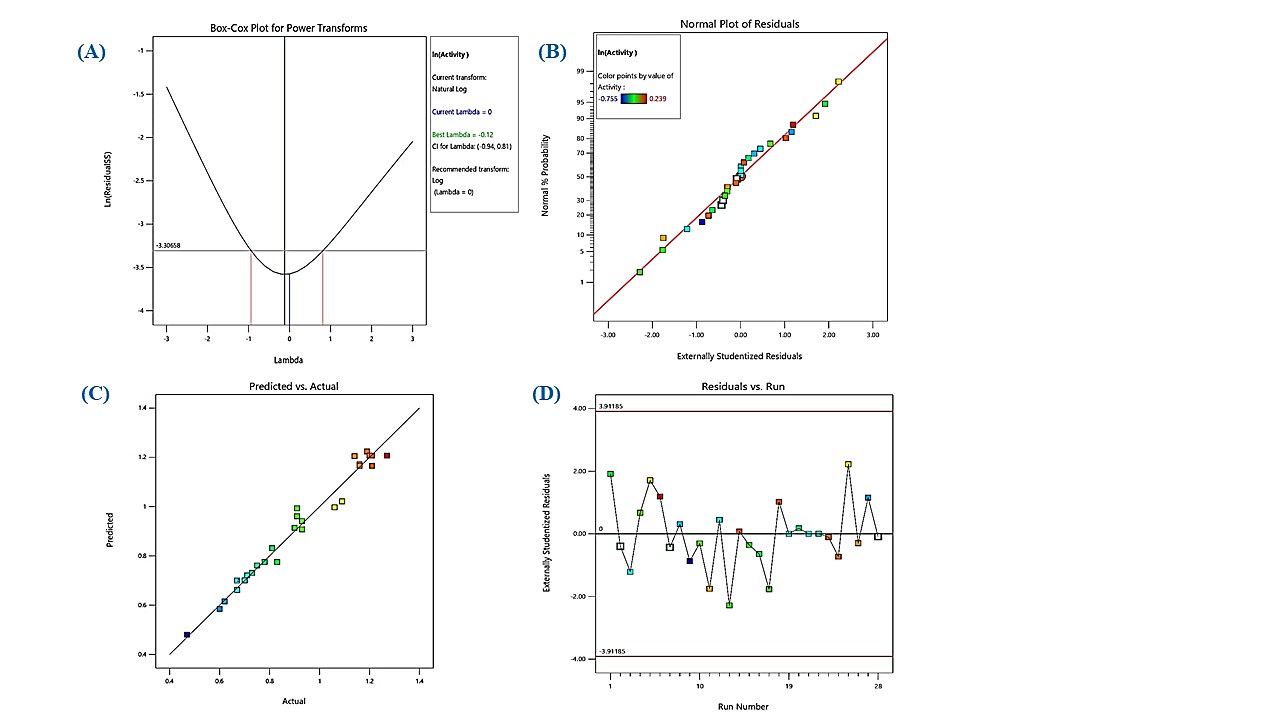


**Figure S4.** Model diagnostic graphs: **(A)** Box-Cox Plot, **(B)** normal probability plot of residuals, **(C)** predicted versus actual plot, **(D)** residuals versus run number plot


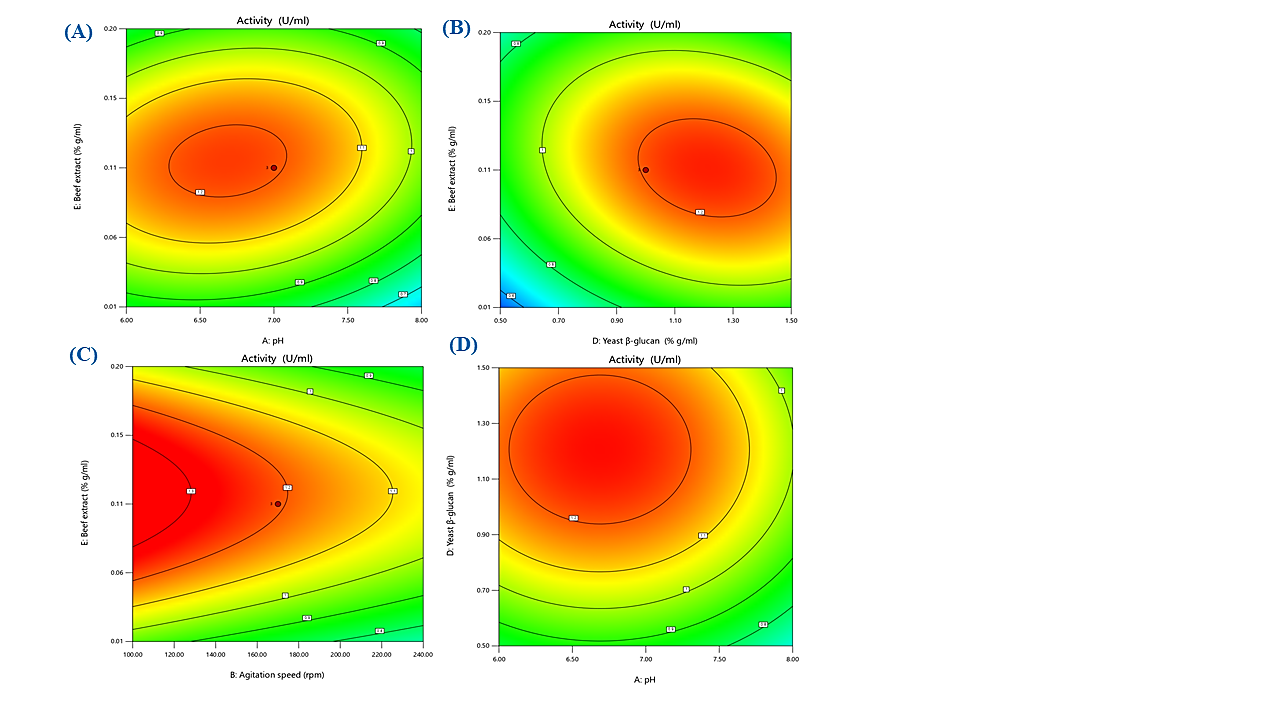


**Figure S5.** Contour plots showing interactions between: **(A)** pH and beef extract concentration (AE), **(B)** yeast β-glucan and beef extract concentrations (DE), **(C)** Agitation speed and beef


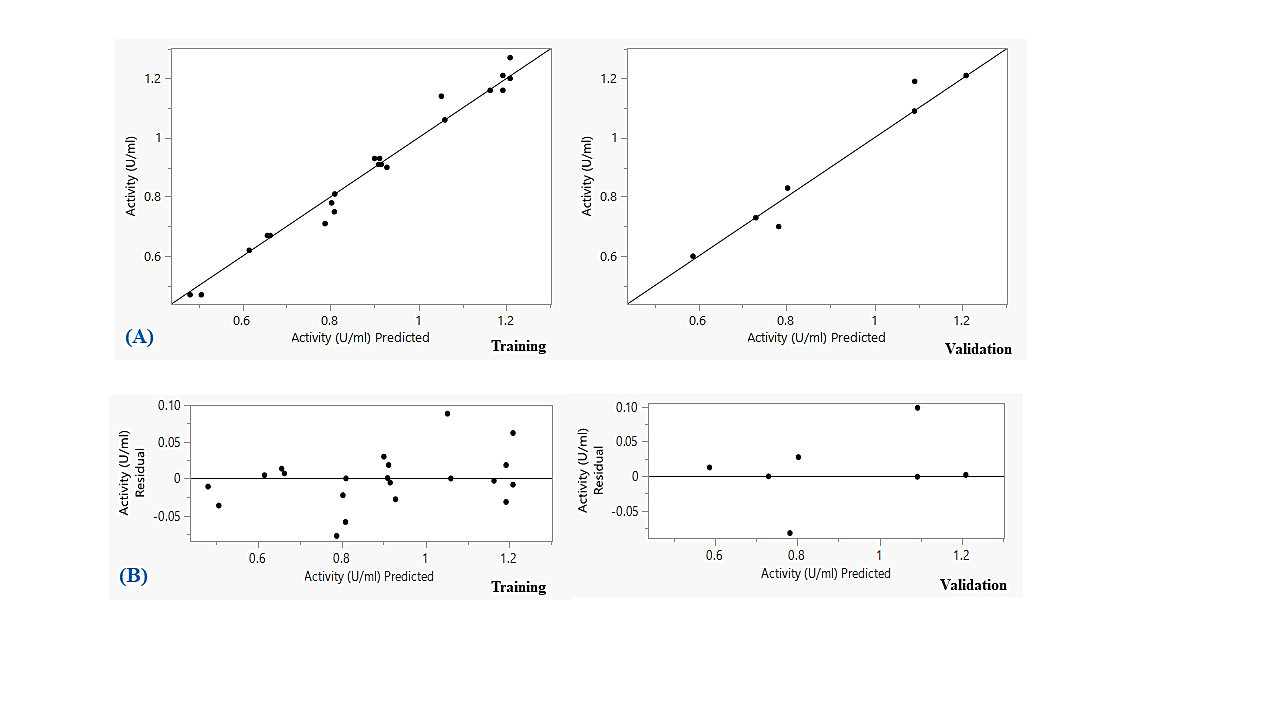

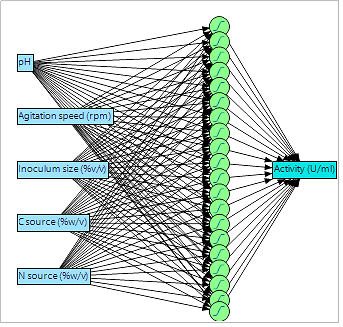


**Figure S7. (A)** The predicted versus actual plot, **(B)** The residual versus actual plot for the ANN model performance over the training and validation sets.

**Figure S6.** The final architecture (5-20-1) of the artificial neural network that best predicts the production of β-glucanase enzyme by S.albogriseolus S13-1.


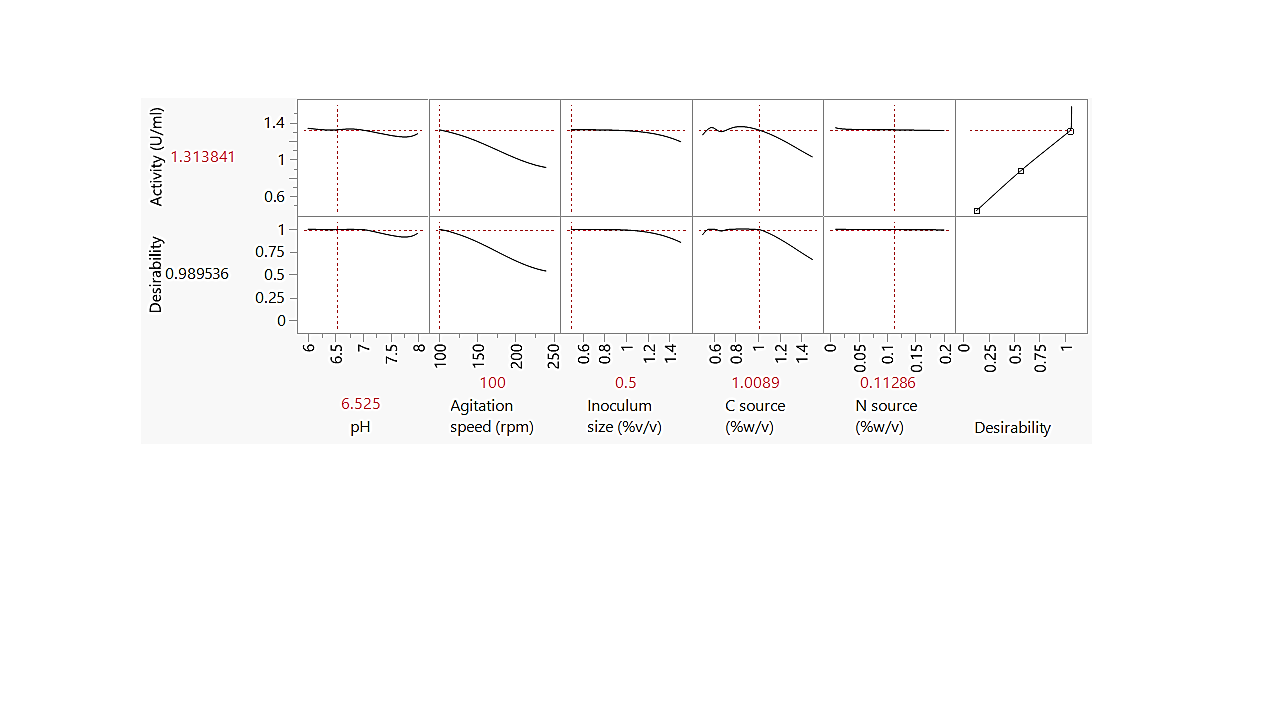


**Figure S8.** The desirability function and the optimal predicted value of β-glucanase production by S. Albogriseolus S13-1.


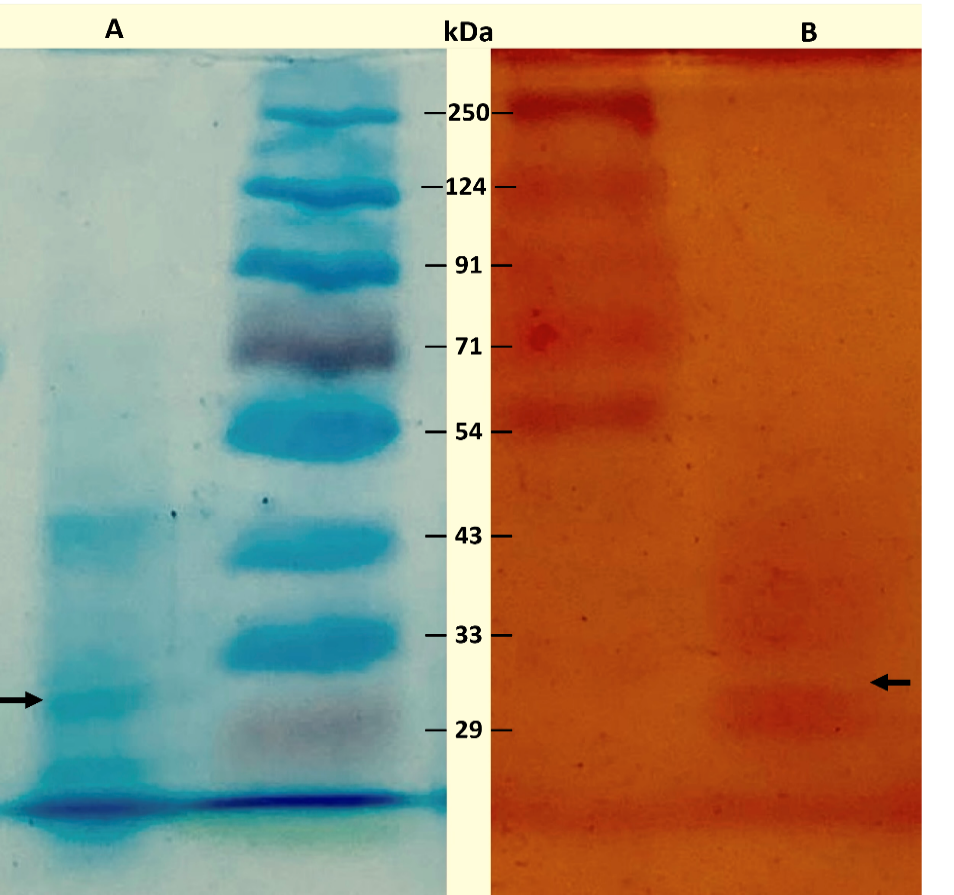


**Figure S9.** SDS-PAGE (A) and zymogram analysis (B) of partially purified β-glucanase


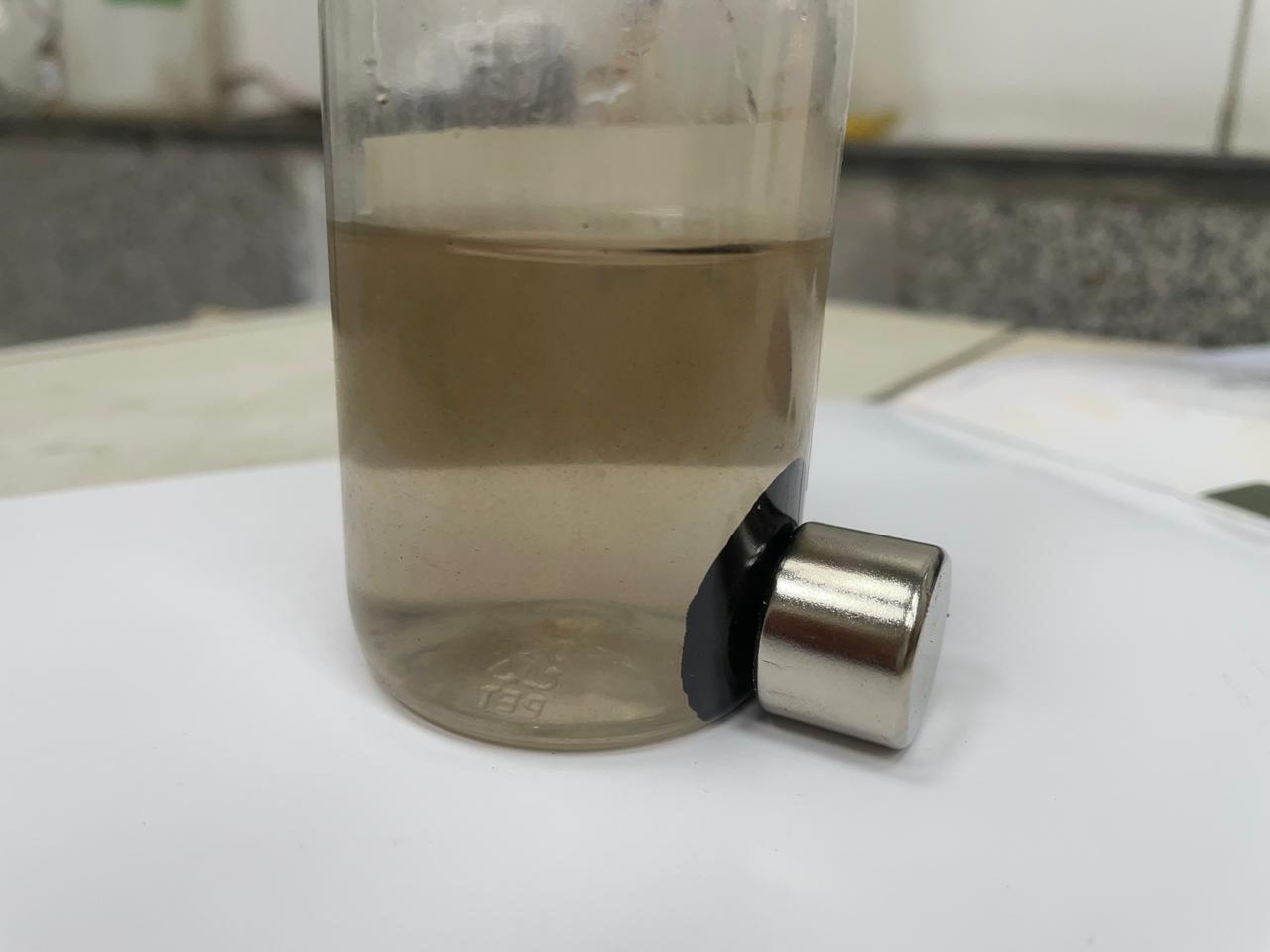

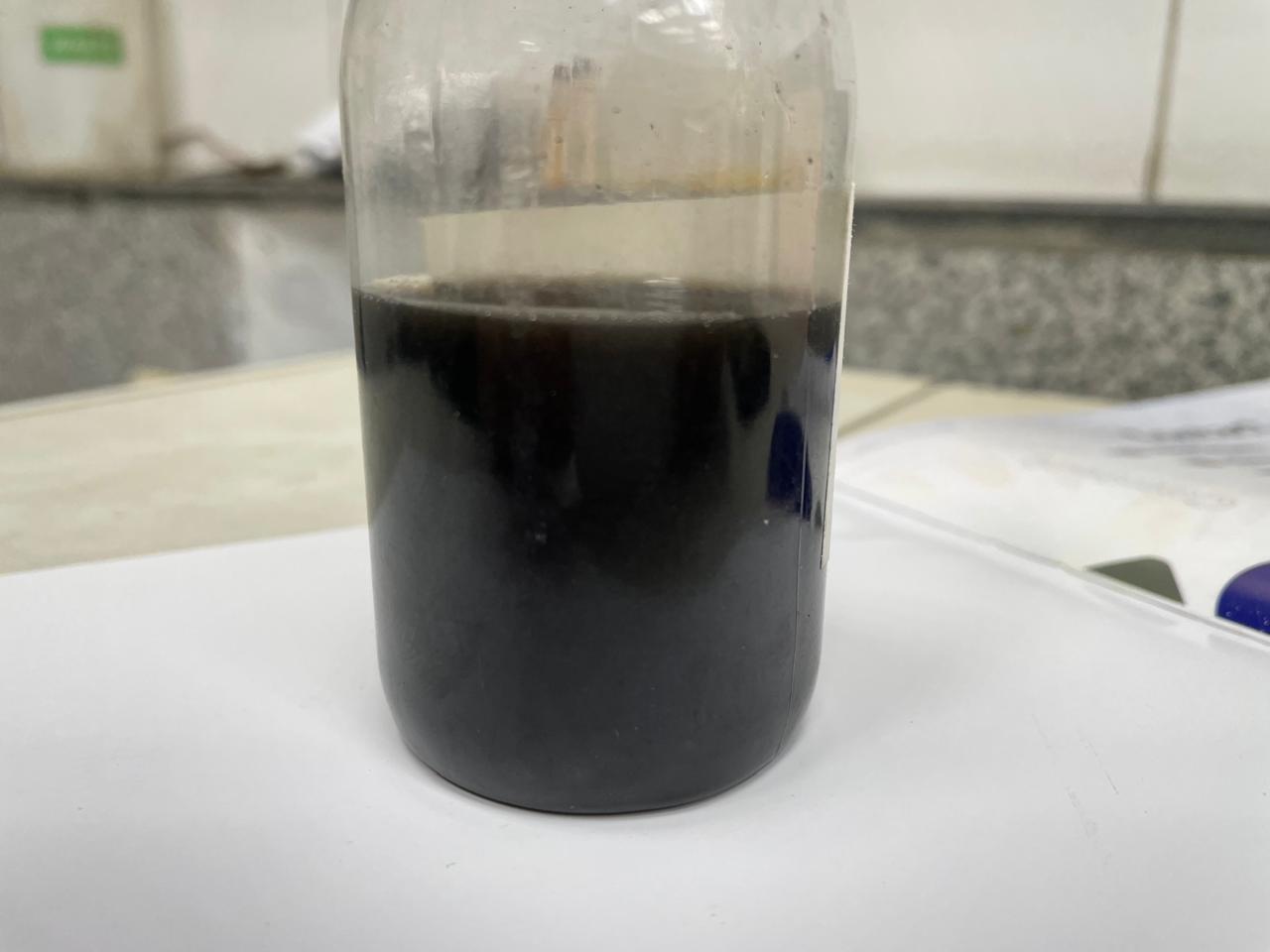


**B**

**Figure S10.** Dispersion of chitosan iron oxide microparticles (A), magnetic attraction of the chitosan iron oxide microparticles to external magnetic field (B).

**A**
